# Differential Site Accessibility Mechanistically Explains Subcellular-Specific *N*-Glycosylation Determinants

**DOI:** 10.3389/fimmu.2014.00404

**Published:** 2014-08-25

**Authors:** Ling Yen Lee, Chi-Hung Lin, Susan Fanayan, Nicolle H. Packer, Morten Thaysen-Andersen

**Affiliations:** ^1^Department of Chemistry and Biomolecular Sciences, Biomolecular Frontiers Research Centre, Macquarie University, Sydney, NSW, Australia

**Keywords:** *N*-glycosylation, solvent accessibility, *N*-glycome, subcellular location, glycoproteome, glycosylation site, *N*-glycan, glycoprotein

## Abstract

Glycoproteins perform extra- and intracellular functions in innate and adaptive immunity by lectin-based interactions to exposed glyco-determinants. Herein, we document and mechanistically explain the formation of subcellular-specific *N*-glycosylation determinants on glycoproteins trafficking through the shared biosynthetic machinery of human cells. LC-MS/MS-based quantitative glycomics showed that the secreted glycoproteins of eight human breast epithelial cells displaying diverse geno- and phenotypes consistently displayed more processed, primarily complex type, *N*-glycans than the high-mannose-rich microsomal glycoproteins. Detailed subcellular glycome profiling of proteins derived from three breast cell lines (MCF7/MDA468/MCF10A) demonstrated that secreted glycoproteins displayed significantly more α-sialylation and α1,6-fucosylation, but less α-mannosylation, than both the intermediately glycan-processed cell-surface glycoproteomes and the under-processed microsomal glycoproteomes. Subcellular proteomics and gene ontology revealed substantial presence of endoplasmic reticulum resident glycoproteins in the microsomes and confirmed significant enrichment of secreted and cell-surface glycoproteins in the respective subcellular fractions. The solvent accessibility of the glycosylation sites on maturely folded proteins of the 100 most abundant putative *N*-glycoproteins observed uniquely in the three subcellular glycoproteomes correlated with the glycan type processing thereby mechanistically explaining the formation of subcellular-specific *N*-glycosylation. In conclusion, human cells have developed mechanisms to simultaneously and reproducibly generate subcellular-specific *N*-glycosylation using a shared biosynthetic machinery. This aspect of protein-specific glycosylation is important for structural and functional glycobiology and discussed here in the context of the spatio-temporal interaction of glyco-determinants with lectins central to infection and immunity.

## Introduction

Significant parts of the human genome and cellular energy are dedicated to produce and regulate protein glycosylation (1). Hence, it is no surprise that this abundant post-translational modification is important in a wide spectrum of biological processes to maintain cellular homeostasis (2). Dysregulation of protein glycosylation is a cause and/or effect of numerous pathological conditions including, but not limited to, congenital disorder of glycosylation (3), cystic fibrosis (4), inflammation (5), auto-immunity (6), and cancer (7). The extracellular location of secreted and cell-surface-tethered proteins carrying *N*-linked glycosylation is ideal for facilitating molecular interactions with the surrounding environment (8). Intracellular functions of *N*-glycoproteins are also known (9, 10). The terminal determinants of host *N*-glycans (so-called “self” and “altered self” in disease) are recognized by endogenous and exogenous glycan-binding proteins commonly called lectins. Interactions between lectins and *N*-glycans are central in innate and adaptive immunity (11). Important examples include the C-type lectins, which may be crudely divided into lectins having affinity for α-mannose/α-fucose-terminated *N*-glycans including dendritic cell-specific intercellular adhesion molecule-3-grabbing non-integrin (DC-SIGN), macrophage mannose receptors and Langerin (12), and lectins having affinity for galactose/GalNAc terminating glycans such as macrophage galactose lectin and DC-asialoglycoprotein receptor (13, 14). In addition, siglecs (I-type lectins) and galectins (S-type lectins) are important for facilitating a functional immune response (15).

The human *N*-glycosylation biosynthetic machinery is relatively well understood (16, 17). In brief, the synthesis is initiated by the transfer of common immature glycan precursors i.e., Glc_3_Man_9_GlcNAc_2_ to conserved sequons (NxT/S, x ≠ P) on translocating polypeptide chains. The glycan precursor is then remodeled through sequential trimming and elongation by specific glycosidases and glycosyltransferases located in the endoplasmic reticulum (ER) and the *cis*-, medial, and *trans*-Golgi, respectively. This series of enzymatic processes first results in the trafficking *N*-glycoproteins being comprised of attached high-mannose-type *N*-glycans, which progresses to the hybrid- and complex-type stage if sufficient interactions with the processing enzymes occur (17). The Golgi-based *N*-glycan processing, including the formation of glycan types and the addition of terminal determinants such as α-fucosylation and α-sialylation, occurs on maturely folded glycoproteins (18, 19). An extensive and reproducible repertoire of *N*-glycans is usually present on a given glycosylation site (20). This *N*-glycan microheterogeneity on proteins results from incomplete processing by the multiple competing enzymatic reactions that can be influenced by cellular factors including the availability of nucleotide sugars, glycosylation enzyme activity, and glycoprotein trafficking time through the biosynthetic machinery. Such cellular factors contribute to cell- and tissue-specific *N*-glycosylation (21). Importantly, the structures of the individual glycoproteins trafficking through the glycosylation machinery dramatically influence the degree of *N*-glycan processing creating protein- and site-specific *N*-glycosylation (22). By thorough literature-based curation of published site-specific glycoprofiling data of mammalian *N*-glycoproteins, we recently confirmed that several structural features including glycan type formation, α1,6-(core) fucosylation, and β1,4/6-GlcNAc branching of *N*-glycans are strongly correlated with the solvent accessibility of the glycosylation sites of maturely folded glycoproteins (19). As such, extensive *N*-glycan processing was observed for proteins displaying solvent accessible glycosylation sites relative to spatially hidden sites. Thus, differential site accessibility can explain how glycoproteins produced simultaneously in the same cell, and even sequons on the same glycoproteins, can present widely different *N*-glycan structural repertoires.

Considering the importance of protein- and site-specific *N*-glycosylation in many aspects of glycobiology including glyco-immunology, we here seek to further explore this feature in the context of the multiple subcellular glycoproteomes that traffic through the shared glycosylation machinery in the secretory pathway of human cells, yet end up at different cellular locations. Due to the functional implications of both intra- and extracellular *N*-glycoproteins, we focus on the secreted, cell-surface, and intracellular glycoproteomes, the latter fraction largely represented by microsomal proteins (23). Understanding, how the subcellular glycoproteomes are generated and regulated under normal and altered physiological conditions of the cell is valuable to the understanding of their involvement in immune biology. Recent analytical developments in glycomics (24–27) and glycoproteomics (28–31) have, together with more conventional proteomics, enabled sensitive, and detailed system-wide investigations of the regulation of protein *N*-glycosylation in immunity (32).

Using LC-MS/MS-based glycomics and proteomics on multiple subcellular fractions from a panel of human cell lines displaying diverse cellular characteristics, we here document that human cells have developed a general mechanism to reproducibly generate vastly different *N*-glycan determinants on their differently located subcellular glycoproteomes that trafficked simultaneously through a shared biosynthetic machinery. We provide evidence that the subcellular-specific protein *N*-glycosylation arises from differential solvent accessibilities of the glycosylation sites of maturely folded glycoproteins that localize to different subcellular compartments following the glycan processing. This aspect of protein-specific glycosylation is discussed here in the context of immunity and infection due to the crucial role of endogenous and exogeneous lectins recognizing exposed self, and altered self, glyco-determinants to facilitate the functional immune response.

## Materials and Methods

### Cellular origin, culture conditions, and doubling time

Multiple human cells showing diverse geno- and phenotypical characteristics were used to demonstrate the general nature of the cellular mechanisms observed in this study. Human mammary epithelial cells (HMEC) were purchased (product # CC-2551, Lonza). Human breast epithelial cell lines MCF10A, MCF7, SKBR3, MDA-MB-157 (MDA157), MDA-MB-231 (MDA231), and HS578T as well as a human colon cancer epithelial cell line SW480 were obtained from American Type Culture Collection (Manassas, VA, USA). HMEC was grown in HuMEC Ready Media (Invitrogen). MCF10A was cultured in DMEM/F12 with the addition of 5% horse serum (Invitrogen), 20 ng/mL epidermal growth factor (EGF) (Invitrogen), 0.5 μg/mL hydrocortisone (Sigma), 100 ng/mL cholera toxin (Sigma), and 8 μg/mL insulin (Invitrogen). Other cell lines were grown in RPMI (Sigma) supplemented in 5% fetal bovine serum (FBS) (Invitrogen), 10 mM glutamine (Invitrogen), and 10 μg/mL insulin. Cells were maintained at 37°C in 5% CO_2_ for all experiments. The breast cell lines were grown in triplicates to ~80% confluence and washed at least four times with ice-cold phosphate buffered saline (PBS) to remove traces of FBS and incubated in serum-free media at 37°C in 5% CO_2_ for 48 h prior to subcellular fractionation.

To measure the cellular doubling times of the breast cell lines, cells were seeded at 1.3 × 10^4^ cells/mL/well in six-well plates and incubated overnight at 37°C in 5% CO_2_. Cells were counted every 24 h over a four-day period using a cell counter (Bio-Rad). The doubling time for each cell line was determined from their exponential growth phase. For overview of the investigated cells and associated data, see Table S1 in Supplementary Material.

### Collection and preparation of subcellular glycoproteomes from breast cell lines

The *secreted* subcellular glycoproteomes were collected by sampling 30 mL of serum-free culture media followed by centrifugation at 2,000 × *g* to pellet any floating cells. The supernatants were concentrated and buffer exchanged into PBS (1×) using 10,000 MWCO Amicon Ultra membranes (Millipore). Proteins were then precipitated with nine volumes of acetone overnight at −20°C. The pellets were stored at −80°C until further analysis.

The *cell-surface* subcellular glycoproteomes were isolated from MCF7, MDA468, and MCF10A breast epithelial cell lines using a commercial biotinylation kit (product # 89881, Pierce) to specifically biotinylate the cell-surface glycoproteins. The protocol supplied by the manufacturer was followed. Briefly, monolayers of cultured cells grown in 75 cm^2^ culture flasks were washed three-times with PBS (1×) before incubation in EZ-Link sulfo-NHS-SS-biotin in ice-cold PBS (1×) for 30 min at 4°C on a rocking platform. The labeling reactions were terminated and the biotinylated cells were washed and collected by scraping in Tris-buffered saline (TBS) (1×), followed by centrifugation at 500 × *g* for 3 min. The supernatants were discarded and the cell pellets were disrupted in manufacturer-provided lysis buffer by ultra-sonication using five 1-s bursts with a Sonifier 450 (Branson Sonifier, Wilmington, NC, USA). The cell lysates were centrifuged at 10,000 × *g* for 2 min at 4°C. Solubilized biotinylated cell-surface proteins in the clarified supernatants were isolated using NeutrAvidin Agarose. Cell-surface-bound proteins were eluted using 50 mM DTT and precipitated with acetone overnight at −20°C. The pellets were stored at −80°C until analysis.

The *microsome* (total membrane) subcellular glycoproteomes were obtained by first removing serum-free media, thoroughly washing cells with PBS (1×), and harvesting cells in 25 mM Tris-HCl pH 7.4, 150 mM NaCl, 1 mM EDTA containing a protease inhibitor cocktail (Roche Diagnostics). The cells were ultra-sonicated on ice for three rounds of 10-s bursts using a Sonifier 450 and centrifuged at 2,000 × *g* for 20 min at 4°C to remove intact cells and nuclei. The supernatants were ultra-centrifuged at 120,000 × *g* for 80 min after which the supernatants were discarded. The microsomal membrane pellets were washed twice with ice-cold 0.1 M sodium carbonate and resuspended in 25 mM Tris-HCl pH 7.4, 150 mM NaCl, and 1% (v/v) Triton X-114. Samples were phase partitioned by incubation at 37°C for 20 min, followed by 1,000 × *g* centrifugation for 10 min. The upper aqueous layer was carefully removed and nine volumes of ice-cold acetone were added to the lower detergent phase and incubated overnight at −20°C to precipitate the proteins. The pellets were stored at −80°C until further analysis.

The protein concentrations of the subcellular fractions were measured using Bradford reagents (Sigma). Equal protein amounts were precipitated in the three subcellular fractions and the resulting pellets were solubilized in 8 M urea for spotting on PVDF membranes for *N*-glycome profiling or in NuPAGE LDS sample buffer for gel electrophoresis prior to proteome profiling.

### Subcellular fractionation of human colon cancer cell lines

SW480 cells (5 × 10^7^) were washed twice with homogenization buffer (20 mM HEPES, pH 7.5, and 0.25 M sucrose). Cell pellets were resuspended to a final volume of 2 mL in homogenization buffer and lysed using an Ultra-Turrax disperser (Ika). After a low speed centrifugation at 1,000 × *g* for 10 min, the supernatant was collected as the post-nuclear fraction (PNF). The PNF was subjected to ultracentrifugation at 30,000 rpm for 1 h in a SW41Ti rotor (Beckman Coulter) to pellet the microsome. ER and Golgi-enriched membranes were prepared as described (33). Briefly, 1 mL of PNF (usually 2.5–3 mg protein) was adjusted to 1.4 M sucrose by adding 2 mL of 2 M sucrose. A discontinuous sucrose gradient was made by sequentially loading 1.5 mL of 1.6 M sucrose, 3 mL PNF in 1.4 M sucrose, 3 mL of 1.2 M sucrose, and 3 mL of 0.8 M sucrose. All sucrose solutions contained 20 mM HEPES pH 7.5. Ultracentrifugation was conducted at 28,500 rpm for 2 h in a SW41Ti rotor. Enriched-Golgi membranes were harvested at the 0.8 M/1.2 M interface. Enriched ER membranes were harvested from the 1.4 M layer. The collected ER and Golgi membranes were diluted by homogenization buffer to reduce concentration of sucrose and subsequently pelleted by ultracentrifugation at 30,000 rpm for 1 h in a SW41Ti rotor. Pelleted ER- and Golgi-enriched membranes were resuspended in 8 M urea and protein concentrations were determined by BCA assays (Pierce).

### Release and preparation of *N*-glycans from subcellular glycoproteomes

*N*-glycans were released from ~20 μg secreted proteins, 50 μg cell-surface proteins, and 50 μg microsome membrane proteins as previously described (27). Briefly, protein mixtures were immobilized on methanol-activated PVDF membranes (Millipore) and allowed to dry overnight. Membrane-bound proteins were incubated with 2.5 U PNGase F (*Flavobacterium meningospeticum*, Roche) for 16 h at 37°C to ensure complete release of *N*-glycans. Released *N*-glycans were incubated with 100 mM ammonium acetate (pH 5) for 1 h at RT and subsequently dried by vacuum centrifugation. Reduction of *N*-glycans was performed with 20 μL 1 M sodium borohydride (Sigma) in 50 mM potassium hydroxide (Sigma) for 3 h at 50°C. Reactions were quenched with 2 μL glacial acetic acid. Dual desalting was performed in micro-SPE formats using strong cation exchange/C_18_ and carbon columns (27). Desalted *N*-glycans were eluted from the carbon columns with 20 μL 40% acetonitrile (ACN) containing 0.1% (v/v) trifluoroacetic acid and dried by vacuum centrifugation (34). Samples were stored at −80°C if not analyzed immediately.

### Digestion and preparation of peptide mixtures from subcellular glycoproteomes

The subcellular glycoproteomes of the breast cells (~50 μg protein/fraction) i.e., secreted, cell surface, and microsomes and of colon cells (~10 μg protein/fraction) i.e., microsome and ER- and Golgi-enriched membrane fractions were reduced and alkylated and subsequently in-gel (breast cells) or in-solution (colon cells) digested. Prior to in-gel digestion, samples were loaded in 10 μL NuPAGE LDS buffer and separated on 4–12% Bis-Tris PAGE gels (Invitrogen). Electrophoresis was performed at 200 V for 50 min. After separation of proteins, gels were fixed in 40% (v/v) ethanol and 10% (v/v) acetic acid for at least 2 h, stained overnight with Coomassie Blue G250 (Bio-Rad) and destained in ultra-pure water (Millipore). In-gel trypsin digestion of all samples was performed from eight equal sized gel fractions. Each fraction was sliced into 1 mm pieces and placed in a 96-well plate. The gel pieces were destained with 50% (v/v) ACN in 50 mM ammonium bicarbonate until clear, dehydrated in 100% (v/v) ACN, and dried. Sequence-grade porcine trypsin (Promega) (1:30 enzyme/substrate, w/w) was used to digest the proteins overnight at 37°C. Tryptic peptide mixtures were then collected and two rounds of gel extractions of peptides were performed with 2% (v/v) formic acid in 50% (v/v) ACN and 50 mM ammonium bicarbonate. The extracts were combined, peptide mixtures dried by vacuum centrifugation, redissolved in 10 μL 0.1% (v/v) formic acid, and desalted as described below. For in-solution digestion, samples were diluted to <1 M urea (final concentration) and trypsinized (sequence-grade porcine trypsin, 1:40 enzyme/substrate, w/w) overnight at 37°C. Following proteolysis, the peptide mixtures were acidified by adding formic acid to a final concentration of 0.1% (v/v). Desalted of peptide mixtures were performed using self-packed C_18_ SPE tips. Briefly, C_18_ tips were washed three-time with 20 μL 100% ACN, three-times with 20 μL 50% (v/v) ACN in 0.1% formic acid, and equilibrated with 50 μL 0.1% (v/v) formic acid. After sample loading, tips were washed three-times with 20 μL 0.1% formic acid. Peptides were eluted with 20 μL 60% (v/v) ACN in 0.1% formic acid and 20 μL 90% (v/v) ACN in 0.1% formic acid and dried. The desalted fractions were dried and stored at −80°C until LC-MS/MS.

### LC-MS/MS-based *N*-glycomics

*N*-glycans alditols were separated using a porous graphitized carbon (PGC) LC column [5 μm (particle size) Hypercarb KAPPA, 100 mm (length) × 200 μm (ID), 250 Å (pore size), Thermo Scientific] using an Ultimate 3000 HPLC system (Dionex) connected directly to an ESI-MS/MS HCT Ultra ion trap (Bruker Daltonics). Separation was performed using a binary gradient solvent system made up of solvent A (aqueous 10 mM NH_4_HCO_3_) and solvent B (90% ACN/10 mM ammonium bicarbonate). The flow rate was 2 μL/min and a total gradient of 100 min was programed as follows: 0–2.5% solvent B for 0–13 min; 2.5–17.5% solvent B for 14–48 min; 17.5–50% solvent B for 48–65 min; 50–100% solvent B for 65–75 min; 100% solvent B for 75–80 min; back to 0% solvent B for 80–85 min, and 100% solvent A equilibration for 15 min. Settings for the MS/MS were as follows: drying gas flow: 6 L/min; drying gas temperature: 300°C; nebulizer gas: 12 p.s.i.; skimmer: −40.0 V; trap drive: −99.1 V; and capillary exit: −166 V. Smart fragmentation was used with start- and end-amplitude of 30 and 200%, respectively. Ions were detected in ion charge control set at 100,000 ions/scan and with maximum accumulation time of 200 ms. MS spectra were obtained in negative ion mode with three scan events: a full scan (*m/z* 400–2,200) at a scan speed of 8,100 *m/z/*s and data-dependent MS/MS scans after CID fragmentation of the top two most intense precursor ions with an absolute intensity threshold of 30,000 and a relative intensity threshold of 5% relative to the base peak. Dynamic inclusion was inactivated to ensure MS/MS generation of closely eluting *N*-glycan isomers. Precursors were observed mainly in charge states *Z* = −1 and/or −2. Mass accuracy calibration of the mass spectrometer was performed using a well-defined tune mix (Agilent) prior to acquisition. *N*-glycans released from bovine fetuin served as positive controls for the sample preparation and the LC-MS/MS performance. Differences between observed and theoretical precursor and fragment masses were generally <0.2 Da. Three LC-MS/MS technical replicates were performed for the subcellular fractions.

### LC-MS/MS-based proteomics

Three LC-MS/MS technical replicates of the subcellular proteomes of the breast cells were analyzed using a Q-Exactive (Thermo Scientific). Peptide mixtures in 0.1% (v/v) formic acid were loaded onto a C_18_ reversed phase column packed in-house [2.7 μm (particle size) HaloLink Resins, Promega, column dimensions: 100 mm (length) × 75 μm (ID)]. Separation of peptides was performed over a 60 min gradient with the first 50 min of the linear gradient increasing from 0 to 50% in solvent B [0.1% (v/v) aqueous formic acid/100% (v/v) ACN] and then to 85% solvent B for the next 2 min and maintained at 85% for 8 min. The flow rate was constant at 300 nL/min. The Easy-nLC (Thermo Scientific) was connected directly to the nano-ESI source of the Q-Exactive. MS full scans were acquired with resolution of 35,000 in the positive ion mode over *m*/*z* 350–2,000 range and an automatic gain control (AGC) target value of 1 × 10^6^. The top 10 most intense precursor ions were then isolated for MS/MS using higher energy collisional dissociation fragmentation at 17,500 resolution with the following settings: collision energy: 30%; AGC target: 2 × 10^5^; isolation window: *m/z* 3.0; and dynamic exclusion enabled. Precursors with unassigned or *Z* = +1 charge states were ignored for MS/MS selection.

The subcellular proteomes of the colon cells were LC-MS/MS analyzed using a Triple TOF 5600 (ABSciex). Peptides were separated by a nanoLC system (Eksigent) on a C_18_ reversed phase column [ProteCol 100 mm (length) × 150 μm, (ID): 3 μm (particle size), 300 Å (pore size); SGE Analytical Science] with a 90 min gradient from 5 to 40% solvent B [90% (v/v) ACN with 0.1% formic acid] at a constant flow rate of 600 nL/min. The top 10 most intense precursor ions with *Z* = +2, +3, and +4 were selected for MS/MS using CID fragmentation.

### Analysis of *N*-glycome LC-MS/MS data

*N*-glycome raw data for all subcellular glycoproteomes were viewed and manually analyzed using DataAnalysis v4.0 (Bruker Daltonics). Monoisotopic masses were obtained and searched against GlycoMod^1^ to obtain possible monosaccharide compositions, which were subsequently verified manually by *de novo* sequencing of corresponding MS/MS spectra and by taking account of PGC chromatographic retention time. The glycan type and the terminating monosaccharide determinants could unambiguously be identified using this method (27). The relative abundances of the observed *N*-glycans were determined using the ratio of the extracted ion chromatogram (EIC) peak area of each *N*-glycan species over the sum of EIC peak areas of all observed *N*-glycans in the sample. This has been shown to be a reasonably accurate method for relative *N*-glycan quantitation (35). The extent of *N*-glycan processing was measured by evaluating the relative molar proportion of the relative unprocessed species (i.e., immature mono-glucosylated glycans and high-mannose type *N*-glycans) and the processed species (i.e., hybrid, complex, and paucimannose type *N*-glycans) of the total *N*-glycome. In addition, the degree of monosaccharide determinants including α1,2/3/6-mannose, β1,3/4-galactose, α1,3/4/6-fucose, and α2,3/6-sialic acid terminating *N*-glycans were calculated as a relative molar abundance of both the entire *N*-glycome and of the potentially modified *N*-glycan substrates (e.g., complex/hybrid-types). Since multiple determinants may be displayed by a given *N*-glycan, the total summed to more than 100%.

### Analysis of LC-MS/MS-based proteomic data and gene ontology

For breast cell proteomes, raw spectra were converted to .mgf files using Proteome Discoverer Daemon v1.3 (Thermo Scientific) and searched against SwissProt protein database (*Homo sapiens*, 20,279 reviewed entries, November 2013 release) using the Global Proteome Machine (Cyclone). The following search criteria were used: carbamidomethylation was a fixed modification and oxidation and deamidation were variable modifications for methionine and asparagine/glutamine residues, respectively. Mass tolerances of 10 ppm and 0.02 Da were selected for precursor and product ions, respectively, with a maximum of two missed tryptic cleavages.

For colon cell proteomes, MS/MS spectra were extracted by ProteinPilot v4.2 (ABSciex) and searched using Mascot v2.4.0 (Matrix Science) against SwissProt protein database (*Homo sapiens*, 20,253 entries, April 2013 release) using trypsin as the digestion enzyme. Precursor and product ion tolerances were 20 ppm and 0.50 Da, respectively. Oxidation of methionine residues and carbamidomethylation of cysteine residues were used as variable modifications.

Scaffold v4.2.1 (Proteome Software) was used to validate MS/MS-based peptide and protein identifications. Peptides were accepted if they were confidently identified at ≥95.0% probability as evaluated by the local false discovery rate (FDR) algorithm. Proteins were included if they were confidently identified at ≥99.0% probability as assigned by the Protein Prophet algorithm incorporated in the software. Proteins containing shared or similar peptides, and which could not be differentiated based on MS/MS analysis alone, were grouped to satisfy the principles of parsimony. Proteins, which confidently shared identified peptides were grouped into clusters. Proteins were annotated using gene ontology (GO) terms from NCBI. The protein identifications were stringently filtered based on the presence of a minimum of two peptides in all replicates. The relative abundances of proteins were determined by conventional spectral counting and adjusted by taking the polypeptide length into account. Putative *N*-glycoproteins in the proteome of the subcellular fractions were predicted *in silico* based on the presence of one or more sequons (NxT/S, x ≠ P) and a signal peptides (for secreted proteins) and/or transmembrane regions (for cell-surface and microsome proteins) using prediction tools including SignalIP (v4.1) (36), Transmembrane Hidden Markov Model (TMHMM v2.0) (37), PrediSi (38), and Phobius (39). Mitochondrial and nuclear membrane proteins were excluded as these are unlikely to enter the ER–Golgi glycosylation pathway. Ambiguous assignments were manually checked (validated or discarded) with information from Uniprot. Potential sequons were obtained using NetNGlyc (40). These *in silico* prediction tools generated lists of experimentally validated and putative glycoproteins. The 100 most abundant glycoproteins in each subcellular fraction were used to assess glycosylation site accessibility. The contribution of these glycoproteins to the total glycoproteome in each sample was estimated by multiplying the normalized spectral count of the individual glycoproteins with their potential glycosylation sites, a measure termed “sequon-weighted normalized spectral count.”

### Selection of PDB 3D structure for glycosylation site accessibility determination

Three-dimensional protein structures were obtained from the protein data bank (PDB) database^2^. If multiple structures were available for a glycoprotein, the best match to the naturally occurring variant was chosen by considering the following parameters in a prioritized order: (1) high protein sequence coverage and resolution of the 3D structure, (2) source of protein (purified from organism/tissue over artificial expression system), (3) known site-specific mutations, (4) presence of artificial/natural ligands, and (5) oligomerization of the solved 3D structure. The experimentally obtained PDB structures used in this study were all based on X-ray crystallography, Table S2 in Supplementary Material. Where no experimentally determined structures were available (43%), structure homologs were obtained from ProteinModelPortal^3^, Swiss-model repository^4^, or ModBase^5^. High sequence homology was used as a selection criterion when choosing homology model. The average sequence homology for all structures was 67%, which is considered very reliable for homology modeling (41), Table S1 in Supplementary Material. 3D protein structures were viewed with RasMol v2.7.5 (RasWin Molecular Graphics) for visual inspection.

### Glycosylation site accessibility determination from maturely folded glycoproteins

The glycosylation site solvent accessibility was determined by measuring the accessibility to the individual asparagine residues forming the glycosylation sites using NACCESS^6^ (42), an accurate and frequently used solvent accessibility determination program (19, 43–45). NACCESS calculates the atomic accessible area by predicting van der Waal’s interactions when a probe is rolled around on the protein surface (46, 47). The maximum probe size offered by the program (5 Å radius) was used as a default in this study to simulate as closely as possible the accessibility of the glycosylation enzymes to the glycosylation sites. NACCESS produces unit-less and absolute accessibility values as the output format (denoted “arb. units”), which are comparable between glycosylation sites of different glycoproteins (19). Prior to the measurements of site accessibility, any water molecules, sugars, ligands, and other hetero-atoms/molecules, not part of the core polypeptide chain, were removed from the protein surface. Negligible accessibility differences were observed for the “native” and the monomeric form of glycoproteins with quaternary structures (data not shown). Hence, in the case of multimers, glycosylation site solvent accessibilities derived from the monomeric structures were not considered in the analysis.

### Statistical analysis

All relative abundances of *N*-glycans were presented as a percentage out of 100% as mean ± SD. Glycosylation site accessibilities were presented as mean ± SEM to illustrate the potential spread of mean instead of the individual data points, which can be hugely influenced by the (local) accuracy and quality of the PDB structures. To overcome this potential issue of PDB “noise,” relative large numbers of data points (*n*) were needed. Data were analyzed using Prism v6 (GraphPad). One-way ANOVA analysis was performed for statistical comparison between the three subcellular fractions followed by *post hoc* Tukey’s tests. All *p* values were adjusted taking into account the multiple comparisons made and reported as multiplicity adjusted *p* values. *p* < 0.05 was regarded as statistically significant and indicated with “*.” Stronger statistical significance was indicated as follows: **p < 0.01; ***p < 0.001; ****p < 0.0001. Simple linear regression and corresponding correlation coefficients (*R*^2^) were obtained to evaluate the relationship between the degree of *N*-glycan processing in terms of glycan type and expression of terminal glycan determinants and the glycosylation site solvent accessibility.

## Results

### Subcellular-specific *N*-glycosylation of human breast epithelial cells

**Figure 1:**
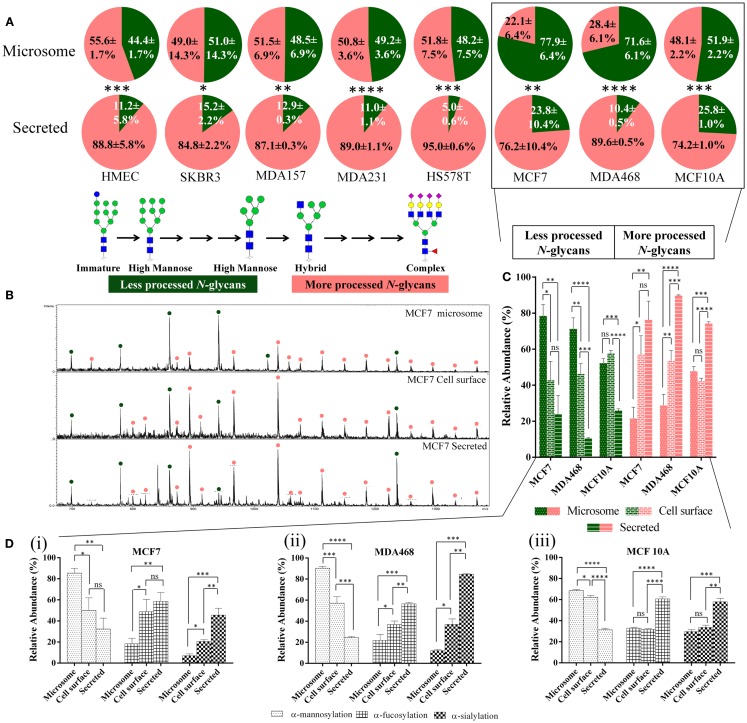
**Secreted glycoproteins display more *N*-glycan type processing than microsomal glycoproteins**. **(A)** The *N*-glycomes of the microsomal (top) and secreted (bottom) proteins of a panel of eight geno- and phenotypically different cultured human breast epithelial cells (i.e., MCF7, SKBR3, MDA157, MDA231, MDA468, HS578T, HMEC, and MCF10A) were profiled, see Table S1 in Supplementary Material for information of investigated cells. The relative molar abundances (mean ± SD) of more processed *N*-glycans comprising the complex, hybrid, and paucimannose type are presented in light red and the less processed *N*-glycans of the immature and high-mannose type in green (inset). Subcellular-specific *N*-glycosylation of boxed cell lines was investigated further in greater detail. **(B)** Summed *m/z* profiles of the *N*-glycomes derived from microsomal (top), cell-surface (middle), and secreted (bottom) proteins of MCF7 cells. Signals corresponding to *N*-glycans have been assigned as less processed (green) or more processed (light red) *N*-glycan types following the same classification as in **(A)**. **(C)** Relative molar distribution (mean ± SD) of more (right, hybrid/complex/paucimannose, light red bars) and less (left, high mannose, green bars) processed *N*-glycan types of the microsomal (dotted bars), cell-surface (brick), and secreted (banded) proteins of MCF7, MDA468, and MCF10A. **(D)** Subcellular-specific distribution of the *N*-glycan determinants. The proportion of terminal α-mannosylation, α-fucosylation, and α-sialylation (non-reducing end) *N*-glycans of the total *N*-glycome (mol/mol %) on the microsome, cell-surface, and secreted glycoproteomes across MCF7 (i), MDA468 (ii), and MCF10A (iii) breast cell lines were determined from the *N*-glycome profiles. *N*-glycans may terminate with multiple monosaccharide determinants making the values sum to more than 100%. For all panels: ns, not significant; *p < 0.05; **p < 0.01; ***p < 0.001; ****p < 0.0001.

Label-free quantitative *N*-glycome mapping of the secreted and microsome (total membrane) subcellular glycoproteomes of a panel of eight cultured human breast cells (i.e., MCF7, SKBR3, MDA157, MDA231, MDA468, HS578T, HMEC, and MCF10A) displaying diverse cellular features showed differential *N*-glycan processing of the two fractions, Figure 1A. The glycoproteins secreted into the cultured media consistently displayed a significantly higher proportion of processed *N*-glycan types (i.e., hybrid, complex, and paucimannose) (74.2–95.0% mol/mol of total *N*-glycome) than the high-mannose-rich microsomal subcellular glycoproteomes (22.1–55.6%, *p* < 0.0001–0.05). Little, if any, correlation between the *N*-glycan processing stage and the cellular doubling time (*R*^2^ = 0.13) or the protein secretion rate (*R*^2^ = 0.35), respectively, was detected of the secreted glycoproteomes across the cell line panel, Figure S1 in Supplementary Material. No correlation was detected between the *N*-glycan processing stage of the microsomal glycoproteins and the cellular doubling time (*R*^2^ = 0.04) or the protein secretion rate (*R*^2^ = 0.01).

In-depth, *N*-glycan profiling of the secreted, microsomal, and cell-surface enriched glycoproteomes was carried out for MCF7, MDA468, and MCF10A cells as representative cells for the breast cell line panel. Differential *N*-glycan processing was evident as exampled by the clear differences seen in the *N*-glycome *m/z* profiles of the three subcellular fractions of MCF7 cells, Figure 1B. The cell-surface glycoproteins derived from MCF7 and MDA468 (but not MCF10A) cells were subjected to more *N*-glycan processing than microsomal proteins (*p* < 0.01–0.05) and all the three cell lines showed significantly increased abundance of the more processed *N*-glycans on the secreted proteins (*p* < 0.0001–0.01), Figure 1C.

### Subcellular-specific distribution of *N*-glycan determinants

To further evaluate the subcellular-specific distribution of common *N*-glycosylation determinants, which may be recognized by different immuno-lectins, terminal α-mannose, α-fucose, and α-sialic acid residues were mapped based on the obtained *N*-glycome profiles, Figure 1D. As expected from the glycan type distribution, terminating α-mannosylation was found to be significantly reduced on the secreted and cell-surface proteins relative to the microsomal proteins. The α-fucosylation, primarily of the α1,6-(core) type, and α2,3/6-sialylation were concomitantly significantly higher in the secreted fractions than in the cell-surface-enriched fraction (with the exception of fucosylation of MCF7) and in the microsomal fraction of all three cell lines. Taking the incomplete subcellular fractionation into account (see “Proteomics- and GO-Based Assessment of Subcellular Fractionation”), we estimate that very little terminal α-mannosylation is present on protein *N*-glycans in contact with the extracellular environment in the investigated cells and that little α-sialylation and α-fucosylation are carried by intracellular (microsomal) *N*-glycoproteins.

### Proteomics- and GO-based assessment of subcellular fractionation

**Figure 2:**
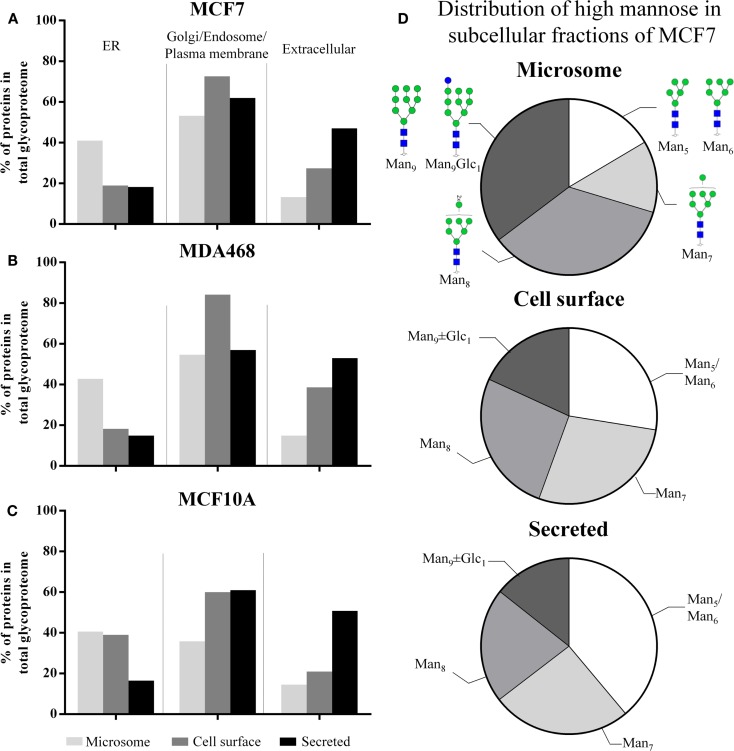
**(A–C)** The subcellular proteomes of MCF7, MDA468, and MCF10A breast epithelial cell lines were mapped according to GO terms into ER, Golgi/endosome/plasma membrane, and extracellular region classifiers. This confirmed enrichment, but not isolation, of cell-surface and secreted proteins in the respective subcellular fractions. In addition, the classification confirmed that the microsomes contained a significant proportion of ER-residing proteins. **(D)** The subcellular distribution of the high-mannose glycan type series on proteins derived from MCF7 into Man_5_, Man_6_, Man_7_, Man_8_, Man_9_ ± Glc_1_, the latter representing immature *N*-glycans normally only associated with intracellular ER *N*-glycosylation. See Figure S2 in Supplementary Material for the subcellular distribution of the high-mannose glycan type series of MDA468 and MCF10A.

In total, 2,297, 2,636, and 2,042 human proteins were identified across the three subcellular fractions in MCF7, MDA468, and MCF10A, respectively. Putative *N*-glycoproteins fulfilling our strict prediction criteria i.e., presence of the following: one or more sequons (NxT/S, x ≠ P); and signal peptides (for secreted proteins); and/or transmembrane regions (for membrane-tethered proteins) comprised significant proportions of the subcellular proteomes (15.7–31.0%), Table S3A in Supplementary Material. The GO terms “ER”, “Golgi/endosome/plasma membrane”, and “extracellular” were used to evaluate the localization/origin of the glycoproteins identified in the subcellular fractions. In agreement with a previous study (23), the GO annotation of the identified proteins showed that the microsomes in general contained a high proportion of ER-residing proteins, Figures 2A–C. Although the proteins are only broadly, and possibly somewhat inaccurately, classified on the basis of GO terms, the trends clearly indicated significant enrichment, although not complete isolation, of the desired proteins in the respective subcellular fractions. The ER-based contribution to the microsome was supported by the fact that a significant proportion of the high-mannose *N*-glycans identified in this fraction were of the immature type i.e., Man_9_ ± Glc_1_ (MCF7: 35.3 ± 0.9%, MDA468: 40.2 ± 2.0%, and MCF10A: 31.8 ± 0.4%, mol/mol of the total high-mannose *N*-glycans), Figure 2D (MCF7 data) and Figure S2 in Supplementary Material (MDA468 and MCF10A data).

To further investigate the intracellular *N*-glycosylation and confirm the presence of ER-rich microsomes, the *N*-glycome and proteome of ER- and Golgi-enriched fractions of human colon epithelial cancer cells (SW480) as prepared by the method of sucrose density gradient centrifugation, were mapped and compared to the microsome profiles derived from the same cells, Figure S3A in Supplementary Material. Quantitative analysis of four reliable and representative markers of the ER (i.e., 78 kDa glucose-regulated protein, protein disulfide bond isomerase, calreticulin, and protein transport protein Sec61 alpha isoform 1) and Golgi (i.e., polypeptide *N*-acetylgalactosaminyltransferase 2, β-1,4-galactosyltransferase 1, Golgi apparatus protein 1, and Golgi membrane protein 1) compartments revealed a high abundance of ER-specific proteins in the ER-enriched fraction, Figure S3B in Supplementary Material. However, there was still a significant presence of ER proteins in the Golgi-enriched and microsome fractions. In contrast, the ER-enriched and microsome fractions were essentially free of Golgi proteins, Figure S3C in Supplementary Material. In line with our breast epithelial cell data, the proteins in the ER-enriched fraction contained a significantly higher degree of high-mannose (Glc_0-1_Man_5-9_GlcNAc_2_) (92%) *N*-glycans than the proteins in the microsome (75%) and the Golgi-enriched fraction (51%). Taken together, the data confirm that the microsomes of human breast and colon epithelial cells predominantly contain ER proteins and that such intracellular proteins mostly carry high-mannose type *N*-glycosylation. Since the Golgi fraction contains few, if any, ER proteins, it becomes clear that the majority of post-ER *N*-glycans are of the complex type.

### Differential Asn site accessibilities explain subcellular-specific *N*-glycosylation

To investigate a possible link between the observed subcellular-specific *N*-glycosylation and protein *N*-glycosylation site accessibility, *in silico* assessment of site accessibility was performed of the identified proteins predicted to be *N*-glycosylated. Due to the laborious and time-consuming approach of determining glycoprotein site accessibility (19), only the most abundant subset of the putative *N*-glycoproteins observed in the subcellular fractions were included in the accessibility assessment. The relative abundances of the individual putative glycoproteins were calculated by a conventional normalized spectral counting strategy; however, the number of sequons of the individual proteins was factored into the calculation to ensure a fair representation of heavily and lightly *N*-glycosylated proteins. We call this term “sequon-weighted normalized spectral counts.” Based on sequon-weighted normalized spectral counts, the 100 most abundant glycoproteins uniquely present in the three subcellular fractions, which, by weight, comprised 70–100% of the individual subcellular glycoproteomes, were used to assess glycosylation site accessibility, Table S3B in Supplementary Material. The solvent site accessibilities were determined using an established approach based on van der Waal interactions of the asparagine residue of the glycosylation sites to solvent (19). 3D-glycoprotein structures (experimental or homology modeled) were available for approximately one-third of the 189, 89, and 183 putative *N*-glycoproteins identified uniquely in the microsome, cell-surface, and secreted fraction, respectively, Figure S4 in Supplementary Material. This yielded site-accessibility datasets covering in total 161 (microsome), 189 (cell-surface), and 236 (secreted) *N*-glycosylation sites from the three cell types.

**Figure 3:**
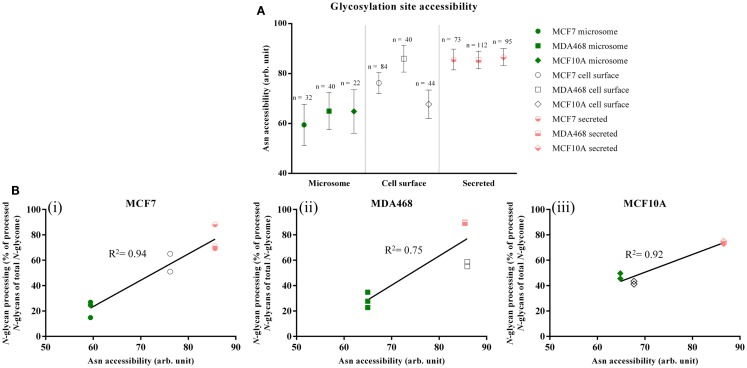
**(A)** Glycosylation site accessibilities (unit-less, arbitrary values, mean ± SEM) of the microsomal (green), cell-surface (white), and secreted (light red) proteins derived from MCF7, MDA468, and MCF10A breast epithelial cell lines. **(B)** Correlation between the site accessibilities and the *N*-glycosylation processing as measured by the more processed *N*-glycan types (hybrid, complex, and paucimannose) as a molar proportion of the total *N*-glycome for the three subcellular fractions. High correlation coefficients (*R*^2^) indicate strong correlation.

Differential site accessibilities were observed for the three subcellular glycoproteomes for all three investigated breast cell lines, Figure 3A (see also Figures S5A–C in Supplementary Material for an alternative representation showing 95% confidence intervals). Glycosylation sites of secreted glycoproteins were significantly more accessible [MCF7: 85.63 ± 35.47, *n* = 73; MD468: 85.44 ± 36.85, *n* = 112; MCF10A: 86.56 ± 33.54 (all unit-less arbitrary values), *n* = 95] than sites on microsomal proteins (MCF7: 59.44 ± 46.58, *n* = 32; MD468: 64.98 ± 46.99, *n* = 40; MCF10A: 64.84 ± 40.97, *n* = 22, *p* < 0.01). In agreement with the *N*-glycomes that carried a mixture of less processed high-mannose and more processed *N*-glycan types, the sites of cell-surface proteins were intermediately accessible: cell-surface sites were either statistically similar in accessibility to the microsomal protein sites (MCF10A: 67.70 ± 37.66, *n* = 44) or similar to the secreted protein sites (MCF7: 76.20 ± 38.13, *n* = 84; MD468: 85.95 ± 34.08, *n* = 40). For all three breast cell lines, the glycosylation site accessibilities were strongly correlated with the *N*-glycan processing as measured by their glycan type (MCF7: *R*^2^ = 0.94; MD468: *R*^2^ = 0.75; MCF10A: *R*^2^ = 0.92), Figure 3B. Higher average glycosylation site accessibility of the secreted and partly also the cell-surface glycoproteins resulted, as such, in more *N*-glycan processing in terms of glycan type formation. Other subcellular-specific *N*-glycosylation signatures including core fucosylation, β-galactosylation, and α-sialylation were found to correlate only weakly or not at all with glycosylation site accessibility upon search for consistent trends across the three different cell lines, Table S4 in Supplementary Material.

## Discussion

### Subcellular-specific protein *N*-glycosylation of human cells

All *N*-linked glycoproteins synthesized by a given cell are processed by a common glycosylation machinery. Despite this shared biosynthetic machinery, we observed that a panel of human breast epithelial cells of different geno- and phenotypes, reproducibly produced subcellular glycoproteomes with distinct *N*-glycosylation signatures. The *N-*glycans attached to proteins enriched from the cell-surface, and in particular the secreted glycoproteins, were significantly more processed with respect to their glycan type (i.e., hybrid/complex/paucimannose) than the predominantly high-mannose type microsomal proteins for all investigated cells. As such, subcellular-specific *N*-glycosylation can be predicted to be a general cellular feature not restricted to the investigated breast epithelial cells. Deeper dissection of the intracellular organelle-specificity of colon cell *N*-glycosylation supported this concept. The capacity of human cells to generate multiple subcellular glycoproteomes displaying specific *N*-glycosylation profiles has, to the best of our knowledge, not been systematically investigated.

The importance of cell-surface *N*-glycosylation for cell–cell and cell–protein interactions has prompted several investigations of the cell-surface (alternatively termed plasma membrane) *N*-glycosylation. High-mannose type *N*-glycans, in particular Man_8–9_ structures, were previously reported to be the dominating features of the plasma membrane of human embryonic stem cells (48) and of cancer cells (49, 50). However, cell lysates and total membrane fractions similar to our microsome preparations were used in these studies suggesting significant contributions from intracellular high-mannose-rich ER-residing *N*-glycoproteins (23). Hence, the actual cell-surface *N*-glycomes in the previous work may not have been accurately captured. Specific cell-surface enrichment methods such as biotinylation labeling strategies used in this study or adhesion-based isolation methods (23) indicate that human cell-surfaces instead are generally decorated with more processed *N*-glycan types.

Of the six cancerous breast cells investigated in this study, only MCF7 and MDA468 displayed predominantly (>70%) high-mannose *N-*glycans of the microsomal proteins. Approximately equal distribution of high-mannose and the more processed *N*-glycan types of microsomal proteins were detected in the remaining four cancerous (SKBR3, MDA157, MDA231, and HS578T) and the two non-cancerous cells (HMEC and MCF10A). In addition, no consistent over-representation of high-mannose *N*-glycans were detected for the secreted proteins derived from the cancerous cell lines relative to the non-cancerous cell lines. Together this indicates that high-mannose *N*-glycosylation is not linked directly to tumorigenesis. Others have associated serum-derived high-mannose *N*-glycoproteins to pathogenesis including cancer and inflammation (5, 51); however, whether these under-processed species are a result of leakage of intracellular glycoproteins as a consequence of cell death or active cellular secretion from intact cells remains to be described. Based on in-depth comparative analysis of the *N*-glycomes derived from secreted proteins of breast and colon epithelial cells of non-cancerous and cancerous nature, we have recently identified several tumor- and sub-type specific *N*-glycosylation signatures amongst the complex *N*-glycans including alterations of sialylation, α1,6-fucosylation, and bisecting β1,4-GlcNAcylation (submitted) (52).

### Site accessibilities mechanistically explain subcellular-specific *N*-glycosylation

We have previously shown that solvent accessibility of the glycosylation site of *N*-glycoproteins is an important factor in generating protein- and site-specific *N*-glycosylation (19). We used literature-based glycoprofiling of more than 100 mammalian glycoproteins produced under different cellular and physiological conditions to establish that site accessibility of maturely folded glycoproteins correlates with *N*-glycan processing features including glycan type, α1,6-fucosylation and β1,4/6-GlcNAc-branching. We emphasized in that study that relatively large datasets were required to compensate for the potential inaccuracy of the individual PDB structures and the relative simplistic solvent accessibility assessment simulating the accessibility of the processing glycosylation enzymes to the protein glycosylation sites.

**Figure 4:**
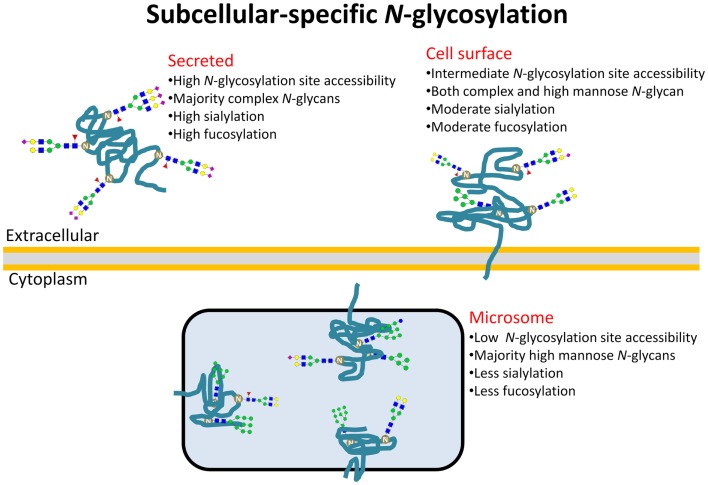
**Subcellular-specific *N*-glycosylation is driven by differential solvent accessibility to the *N*-glycosylation sites on maturely folded glycoproteins**. Consequently, the *N*-glycans of secreted, cell-surface, and microsomal proteins receive high, intermediate, and low *N*-glycosylation processing, respectively, and as a result, display distinct glyco-determinant signatures.

Herein, we used a similar approach using our own *N*-glycosylation data acquired from eight cell lines fractionated into subcellular glycoproteomes to further explore the determining features of site-specific *N*-glycosylation in the context of subcellular localization of proteins. Homogenous cell cultures were an essential tool to ensure that the isolated subcellular glycoproteomes were produced simultaneously under the same physiological conditions of the glycosylation machinery. Although the *N*-glycomes, as expected, varied considerably between the different cell lines, our experimental data not only validated the strong correlation of the *N*-glycan type and the glycosylation site accessibility of maturely folded glycoproteins in agreement with our previously report (19), but also mechanistically explained that subcellular-specific *N*-glycosylation is driven by differences in site accessibilities of the individual glycoproteins ending up at different subcellular destinations, Figure 4. Intracellular (microsome) *N*-glycoproteins receive little glycan processing of the high-mannose intermediates as a result of limited site accessibility, whereas the secreted *N*-glycoproteins are modified almost entirely to more processed *N*-glycan types due to high site accessibilities. As such, *N*-glycan processing may be a targeting signal or a requirement for intracellular (ER–Golgi-residing) glycoproteins to translocate to the surface for cell-surface integration/secretion via vesicles. Keeping in mind there may be many exceptions to the molecular trends presented here, it is tempting to view the glycosylation site accessibility, and, thus, the *N*-glycan type, as a crude predictor of subcellular location of human glycoproteins.

We have previously linked core fucosylation to glycosylation site accessibility (19). Interestingly, glycosylation site accessibility alone could not explain the differential core fucosylation of the subcellular fractionated proteins in our data: the secreted proteins did not have a higher degree of core fucosylation of complex/hybrid-type *N*-glycans than the cell-surface proteins although the secreted proteins had significantly higher accessibilities. This surprising observation may be explained by a possible advantage of the membrane-embedded cell-surface glycoproteins to achieve preferential interaction with the membrane-bound fucosyltransferase 8 (FUT8) facilitating the addition of α1,6-fucose residues to the chitobiose cores of *N*-glycans. Soluble (luminal) glycoproteins may be less likely to interact with FUT8. This explanation is congruent with our previous observation describing FUT8 discrimination of soluble *N*-glycoproteins over membrane *N*-glycoproteins (19). Similar processing preference was not observed for the multiple processing enzymes responsible for the formation of the glycan type. As expected, the glycan modification more distal from the protein surface i.e., β-1,3/4-galactosylation and α2,3/6-sialylation were not found to be correlated with glycosylation site accessibility since the glycosyltransferases most likely have unhindered access to the substrates relatively far from the protein surface. By the same token, we cannot rule out that a more refined accessibility determination approach, which not only takes into account the glycosylation site solvent accessibility, but also the conjugated *N*-glycans (53–56), may expose that other subcellular-specific *N*-glycan features correlate with site accessibility. New developments in glycoproteomics may also support and strengthen these observations by giving more accurate insight into the connectivity of glycosylation of the individual protein carriers (31). Finally, it should be emphasized that although the subcellular glycoproteomes share a common biosynthetic machinery, slightly different trafficking rates and/or routes to their final destinations are factors that may contribute to yield distinct subcellular *N*-glycosylation. Other cellular factors including the glycosylation enzyme activity or the availability of nucleotide donors may also indirectly contribute to subcellular-specific *N*-glycosylation by having differential effects on the individual subcellular glycoproteomes.

### Subcellular-specific glyco-determinants in immunity

The distinct *N*-glycosylation signatures carried by the subcellular glycoproteomes may be functionally important in immunity if we consider the key role of *N*-glycans as mediators for an effective innate and adaptive immune response through their specific interaction with endogenous lectins. In addition, opportunistic pathogens often use exposed *N*-glycan determinants as receptors for adhesion using exogenous lectins (11). The observed subcellular-specific glycosylation is here briefly discussed in the context of glyco-immunity and infection; it is stressed that further empirical evidence is required to validate these proposed relationships.

We found that α-sialylation was a more abundant feature of the secreted *N*-glycoproteins than cell-surface proteins. High sialylation of secreted glycoproteins is essential to mask penultimate galactose residues from being exposed and recognized by asialoglycoprotein receptors, a C-type lectin (12). Thus, the high sialylation of secreted glycoproteins may be a requirement to ensure prolonged circulation half-life. In addition, high sialylation of secreted glycoproteins can act as a strong decoy for the less sialylated cell-surface proteins, to which opportunistic pathogens are known to adhere through sialic acid-recognizing I-type lectins (alternatively termed siglecs) (57, 58). Displaying less-than-complete sialylation of the cell-surface proteins also ensures that a gradient of biological activity toward endogenous siglecs for cellular signaling and endocytosis (59) is maintained through structural diversity, which may confer an immunological advantage to the host cells (60).

The secreted *N*-glycoproteins were over-represented in α1,6-core fucosylation relative to the cell-surface proteins. In line with our previous observations, the higher degree of core fucosylation may serve to either mask hydrophobic patches to regulate stability/solubility of the secreted *N*-glycoproteins (19) or to protect these more exposed proteins from proteolytic degradation in the extracellular environment. It could be speculated that the membrane-embedded nature of cell-surface glycoproteins would make them more stable by not facing solubility issues in their local environment and less vulnerable to proteolytic digestion, thereby having less requirement for steric protection provided by a bulky fucose residue proximal to the protein surface.

We and others have observed that α-mannose is an unusual terminating structural determinant in the extracellular environment (61, 62). This may partly be explained by the intracellular functions of mannose (and glucose) terminating *N*-glycans (16, 17). The presence of several mannose recognizing lectins in the extracellular environment including mannan binding protein (MBP), DC-SIGN, and macrophage mannose receptors may be relevant in the context of apoptosis when mannose terminating *N*-glycoproteins are exposed to the extracellular environment. In particular, MBP is a key player and a first line of defense in innate immunity, enabling phagocytosis of apoptotic cells through its binding to exposed immature or under-processed glycans or to pathogens carrying mannosylated glycoproteins (63, 64). Hiding mannose inside cells under physiological conditions could thus be viewed as being critical to avoiding the unnecessary onset of inflammation and auto-immunity. The presence of extracellular α-mannosylation would, as such, be indicative of pathophysiological conditions. In support of this hypothesis, high-mannose containing glycoforms of intracellular adhesion molecule 1 and EGF receptor on cell-surfaces were shown to contribute to endothelial inflammation (61) and correlated with poor prognosis of various cancers, respectively (61, 62).

It has been noted that the structure and function of the protein *N*-glycome is different within and outside human cells and that these differences may be shaped by evolutionary forces (60). We are the first to systematically investigate and mechanistically explain some aspects of subcellular-specific *N*-glycosylation. We conclude that human cells have developed protein structure-specific mechanisms including differential *N*-glycosylation site accessibilities to generate subcellular glycoproteomes that display distinct *N*-glycosylation phenotypes using a shared biosynthetic machinery. Establishing this relationship is of general significance to glycobiologists and in particular to molecular immunologists due to the functional relevance of *N*-glycan determinants acting as ligands for the spectrum of endogenous lectins involved in facilitating an efficient immune response.

## Conflict of Interest Statement

The authors declare that the research was conducted in the absence of any commercial or financial relationships that could be construed as a potential conflict of interest.

## Supplementary Material

The Supplementary Material for this article can be found online at http://journal.frontiersin.org/Journal/10.3389/fimmu.2014.00404/abstract

## Footnotes

^1^http://web.expasy.org/glycomod/

^2^http://www.rcsb.org/pdb

^3^http://www.proteinmodelportal.org

^4^http://swissmodel.expasy.org

^5^http://modbase.compbio.ucsf.edu/modbase-cgi/index.cgi

^6^http://wolf.bms.umist.ac.uk/naccess/
