# Supplementary material for: Differential Site Accessibility Mechanistically Explains Subcellular-Specific N-Glycosylation Determinants
Source: Front Immunol. 2014 Aug 25;5:404. doi: 10.3389/fimmu.2014.00404 (PMC4142333; doi:10.3389/fimmu.2014.00404)
Supplement: Supplementary file 1 [file Data_Sheet_1.PDF]

Supplementary Material
Differential site accessibility mechanistically explains subcellular-specific *N*-glycosylation determinants

Lee, L.Y., Lin, C-H., Fanayan, S., Packer, N.H., Thaysen-Andersen, M*.

Biomolecular Frontiers Research Centre, Dept. of Chemistry and Biomolecular Sciences, Macquarie University, Sydney, Australia

Running title (Max 5 word): Differential Asn-accessibility generates subcellular-specific *N*-glycosylation

*** Corresponding author**:
Morten Thaysen-Andersen, Ph.D.
Department of Chemistry and Biomolecular Sciences
Macquarie University
NSW 2109, Australia
Phone: +61 2 9850 7487
Fax: +61 2 9850 6192
E-mail: morten.andersen@mq.edu.au

Supplementary Figures

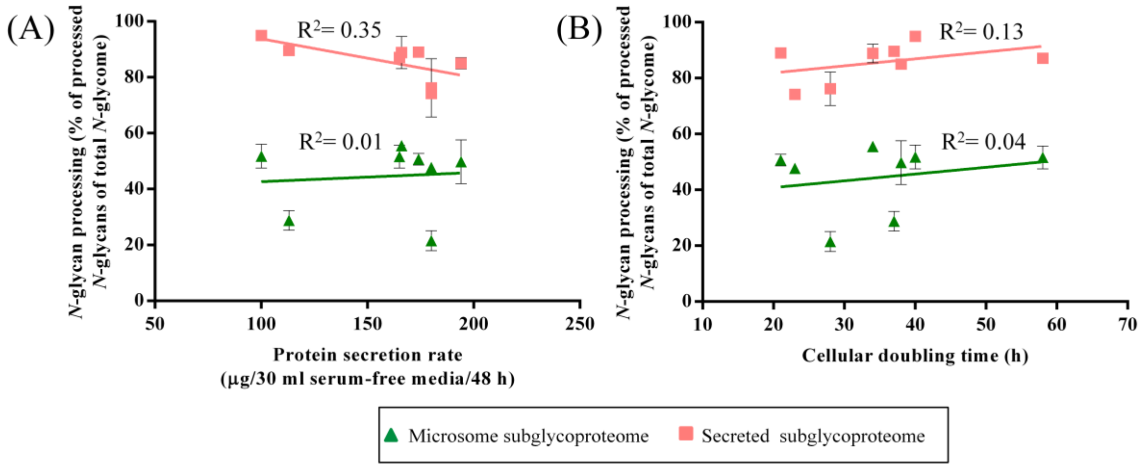

Supplementary Figure 1. **(A)** Secretion rate as measured by the protein concentration (µg / 30 mL serum-free media / 48 h) in the culture media shows weak correlation with the *N*-glycan processing as measured by the molar proportion of more processed *N*-glycan types (i.e. hybrid, complex and paucimannose) of the total *N*-glycome for the secretome (R^2 = 0.35) (light red) but not the microsome (R^2 = 0.01) (green) derived from the investigated panel of human breast cell lines. **(B)** No/negligible correlations were observed between the cellular doubling time (hours) and *N*-glycan processing of either the secretome (R^2 = 0.13) or the microsome (R^2 = 0.04).

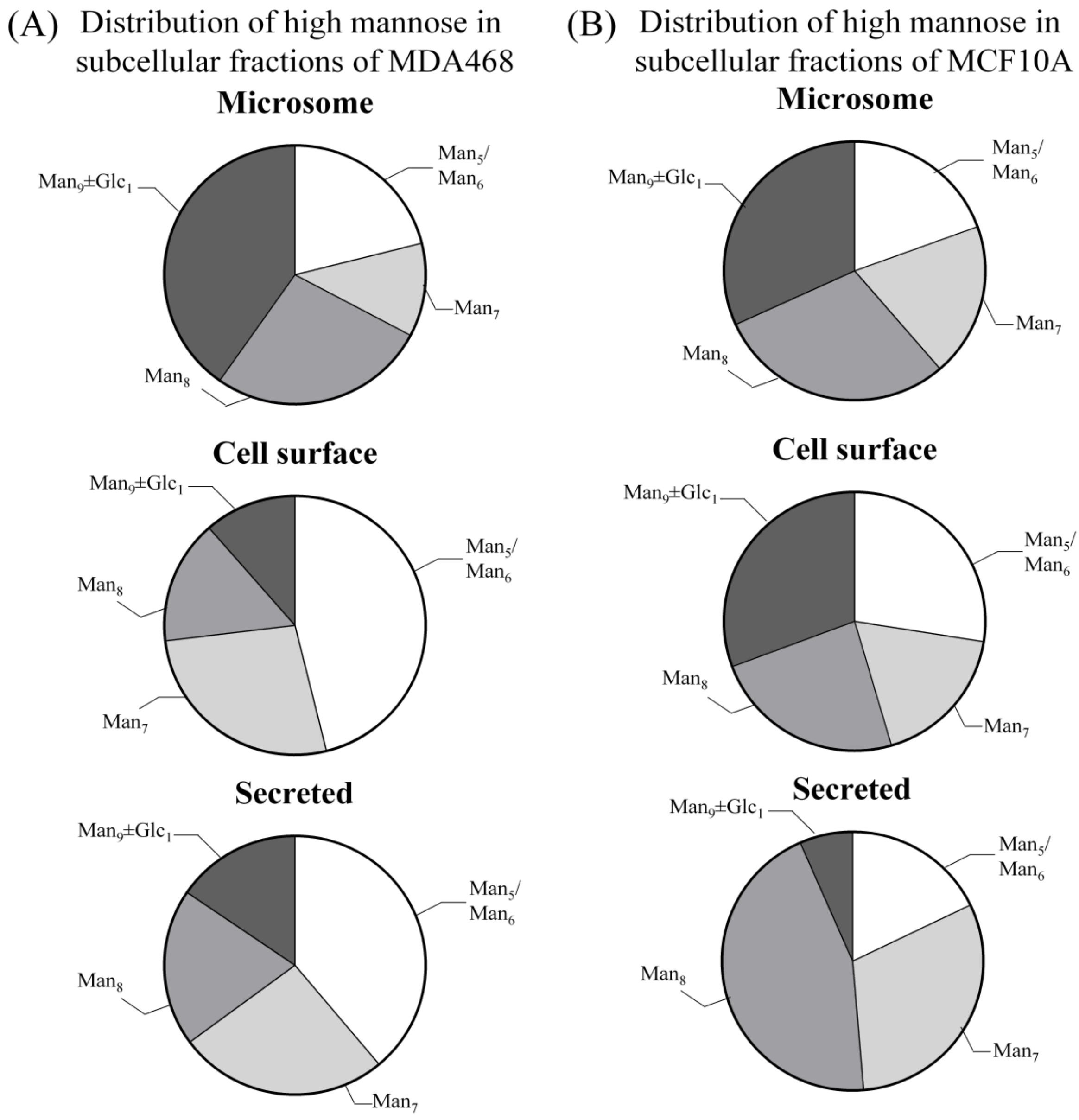

Supplementary Figure 2. The subcellular-specific distribution of the high mannose series of **(A)** MDA468 and **(B)** MCF10A into Man_5, Man_6, Man_7, Man_8, Man_9 ± Glc_1. The latter represents immature *N*-glycans.

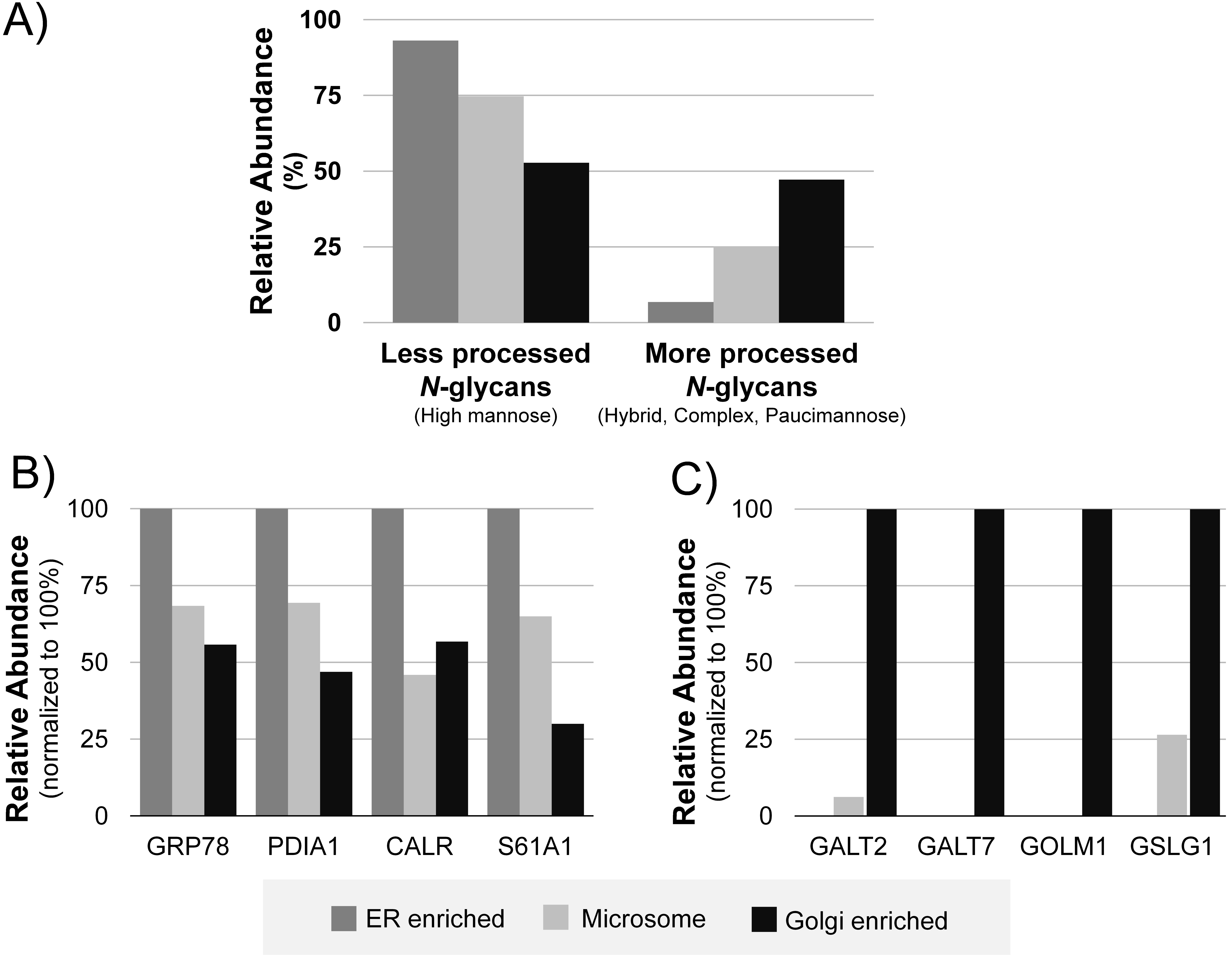

A)
Relative Abundance (%)
100
75
50
25
0
Less processed N-glycans
(High mannose)
More processed N-glycans
(Hybrid, Complex, Paucimannose)
B)
Relative Abundance (normalized to 100%)
GRP78
PDIA1
CALR
S61A1
C)
GALT2
GALT7
GOLM1
GSLG1
ER enriched
Microsome
Golgi enriched

Supplementary Figure 3. **(A)** *N*-glycome mapping of the three subcellular fractions of SW480 i.e. ER-enriched (dark grey bars), microsome (light grey bars) and Golgi-enriched (black bars) fractions. *N*-glycome profiles are presented as the molar distribution of the less processed (left, high mannose type *N*-glycans) and more processed (right, complex/hybrid/paucimannose) *N*-glycan types. **(B-C)** Quantitative LC-MS/MS-based proteomics of the same subcellular fractions confirmed enrichment of the desired proteins by mapping the relative abundances of known ER- (left) and Golgi- (right) protein markers.

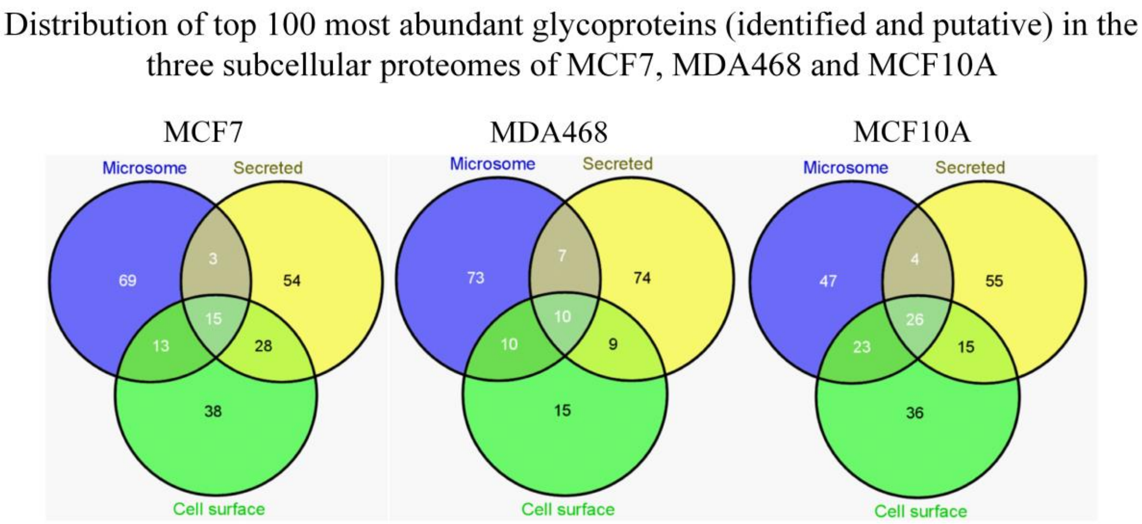

Supplementary Figure 4. The distribution of the top-100 most abundant putative glycoproteins in the three subcellular proteomes of MCF7, MDA468 and MCF10A. The unique proteins identified in each subcellular fraction were used for determining average glycosylation site accessibilities of the subcellular glycoproteomes.

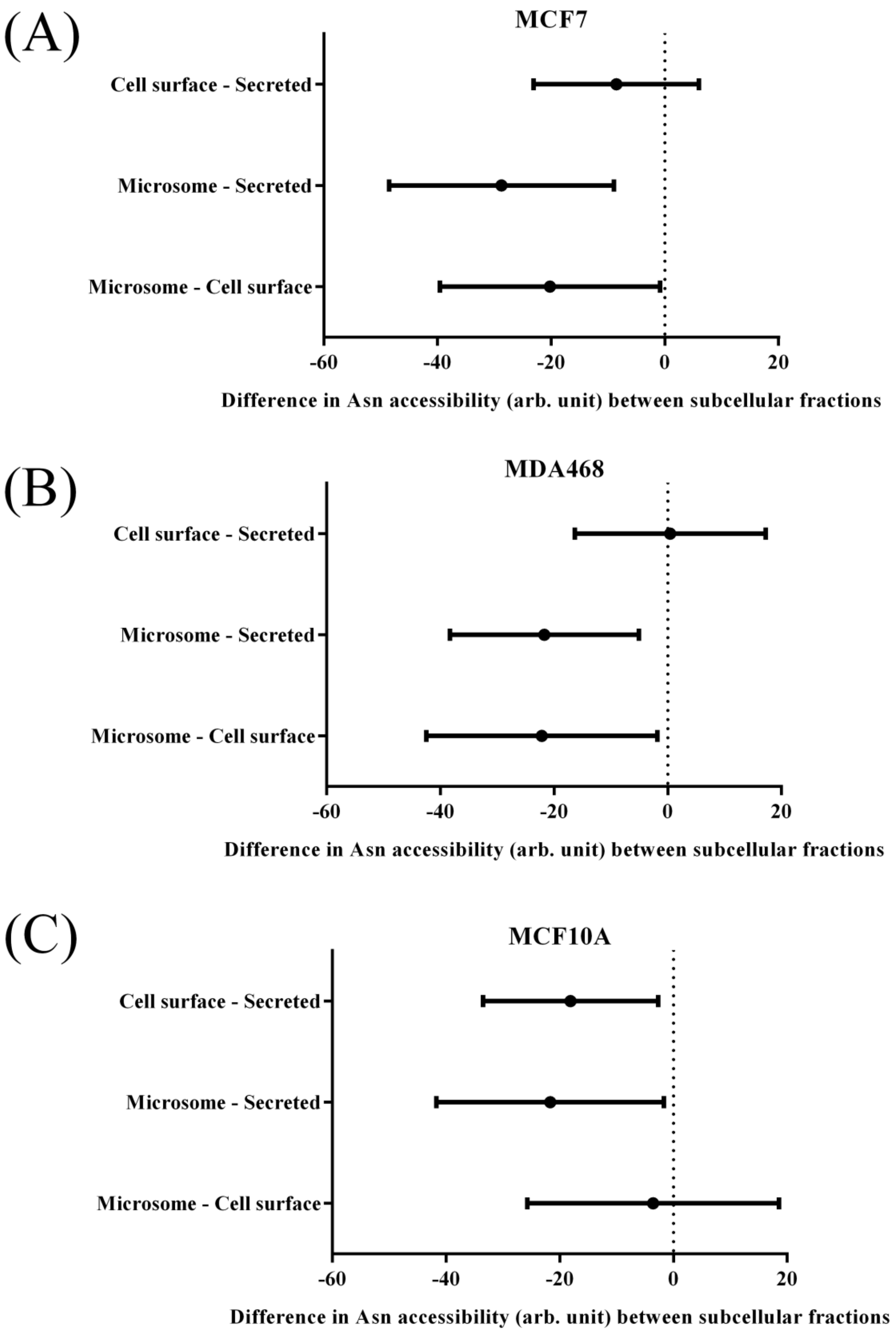

Supplementary Figure 5. The comparison of subcellular-specific glycosylation site accessibilities (unit-less, arbitrary values) in (A) MCF7, (B) MDA468 and (C) MCF10A as measured by one-way ANOVA. The graphs show the 95% confidence intervals for the differences between the means (site accessibility).

Supplementary Tables

Supplementary Table 1.
Overview of investigated panel of human breast cells. Subtype, presence (+) / absence (-) / data not available (NA) of hormone receptors (i.e. estrogen receptor (ER), progesterone receptor (PR), and ERBB2), origin as extracted from ATCC and other supporting literature is listed. Doubling time (hours) and protein secretion rate (µg / 30 mL serum-free media / 48 h) were determined for all cells and plotted against the *N*-glycan type processing of the secreted and microsome subcellular glycoproteomes (see **Supplementary Figure 1**).

**Cell line**	**Sub-type**	**ER / PR/ ERBB2**	**Origin**	**Doubling time*** (h)	**Protein secretion** (µg / 30 mL media / 48 h)	**Viability** (%)
MCF7	Luminal	+/+/-	Human adenocarcinoma	28	180	93
SKBR3	Luminal	-/-/+	Human adenocarcinoma	38	194	97
MDA157	Basal B	-/-/-	Human medullary carcinoma	58	165	92
MDA231	Basal B	-/-/-	Human metastatic adenocarcinoma	21	174	98
MDA468	Basal A	-/-/-	Human metastatic adenocarcinoma	37	113	91
HS578T	Basal B	-/-/-	Human carcinoma sarcoma	40	100	98
HMEC	NA	NA / NA / NA	Human mammary epithelia cells, primary tissue	34	166	95
MCF10A	Basal B	-/-/-	Human fibrocystic disease	23	180	92

Supplementary Table 2

Summary of unique glycoproteins from the three subcellular fractions i.e. secreted, cell surface and microsome subglycoproteome and the solvent accessibility of asparagine residues residing in conserved sequons (mean ± SD).

MCF7 Microsome (Unique)

Acc No	Identified Proteins	Uniprot ID	PDB or Model	%matches	Asn Site	Accessibility	Protein Length	Signal Peptide	Trans-membrane	Glycosylation sites (No)	Glycoprotein	Mol Weight	
P28288	ATP-binding cassette sub-family D member 3	ABCD3_HUMAN	N/A				659	N	Y	1	Y	75 kDa	N = No
Q8N2K0	Monoacylglycerol lipase ABHD12	ABD12_HUMAN	N/A				398	N	Y	1	Y	45 kDa	Y = Yes
O00767	Acyl-CoA desaturase	ACOD_HUMAN	N/A				359	N	Y	3	P	42 kDa	P = Potential
Q9HDC9	Adipocyte plasma membrane-associated protein	APMAP_HUMAN	N/A				416	Y	Y	2	Y	46 kDa	N/A = Not available
Q9HD20	Probable cation-transporting ATPase 13A1	AT131_HUMAN	N/A				1204	N	Y	6	Y	133 kDa	
P54709	Sodium/potassium-transporting ATPase subunit beta-3	AT1B3_HUMAN	Model on 2kdpB	40	Asn124	116.34	279	Y	Y	3	Y	32 kDa	
			Model on 2kdpB		Asn240	78.57							
P16615	Sarcoplasmic/endoplasmic reticulum calcium ATPase 2	AT2A2_HUMAN	Model on 3ar4	84	Asn19	78.53	1042	N	Y	7	P	115 kDa	
			Model on 3ar4		Asn421	10.53							
			Model on 3ar4		Asn589	71.33							
			Model on 3ar4		Asn738	6.43							
			Model on 3ar4		Asn918	46.93							
			Model on 3ar4		Asn962	75.61							
Q93084-2	Isoform SERCA3A of Sarcoplasmic/endoplasmic reticulum calcium ATPase 3	AT2A3_HUMAN	Model on 3ar4a	84	Asn213	48.52	1043	N	Y	3	P	109 kDa	
			Model on 3ar4a		Asn421	11.44							
			Model on 3ar4a		Asn919	36.27							
Q8NHH9	Atlastin-2	ATLA2_HUMAN	Model on 4IDO	73	Asn163	116.43	583	N	Y	2	P	66 kDa	
			Model on 4IDO		Asn204	1.64							
Q6DD88	Atlastin-3	ATLA3_HUMAN	Model on 3q5e	69	Asn173	2.3	541	N	Y	5	P	61 kDa	
			Model on 3q5e		Asn312	11.41							
			Model on 3q5e		Asn432	17.41							
O75110	Probable phospholipid-transporting ATPase IIA	ATP9A_HUMAN	N/A				1047	N	Y	4	P	119 kDa	
P27824	Calnexin	CALX_HUMAN	N/A				592	Y	Y	1	Y	68 kDa	
Q96A33	Coiled-coil domain-containing protein 47	CCD47_HUMAN	N/A				483	Y	Y	1	Y	56 kDa	
P08962	CD63 antigen	CD63_HUMAN	N/A				238	Y	Y	3	Y	26 kDa	
O96005	Cleft lip and palate transmembrane protein 1	CLPT1_HUMAN	N/A				669	N	Y	6	Y	76 kDa	
P52569	Low affinity cationic amino acid transporter 2	CTR2_HUMAN	N/A				658	N	Y	4	Y	72 kDa	
Q53GQ0	Estradiol 17-beta-dehydrogenase 12	DHB12_HUMAN	N/A				312	N	Y	2	P	34 kDa	
Q9UBM7	7-dehydrocholesterol reductase	DHCR7_HUMAN	N/A				475	N	Y	2	P	54 kDa	
Q15125	3-beta-hydroxysteroid-Delta(8),Delta(7)-isomerase	EBP_HUMAN	N/A				230	N	Y	1	P	26 kDa	
Q9BW60	Elongation of very long chain fatty acids protein 1	ELOV1_HUMAN	N/A				279	N	Y	2	P	33 kDa	
Q8N766	ER membrane protein complex subunit 1	EMC1_HUMAN	N/A				993	Y	Y	3	Y	112 kDa	
P33947	Cluster of ER lumen protein retaining receptor 2	ERD22_HUMAN	N/A				212	N	Y	1	P	24 kDa	
Q14534	Squalene monooxygenase	ERG1_HUMAN	N/A				574	N	Y	1	P	64 kDa	
Q969X5	Endoplasmic reticulum-Golgi intermediate compartment protein 1	ERGI1_HUMAN	N/A				290	Y	Y	1	Y	33 kDa	
O94905	Cluster of Erlin-2	ERLN2_HUMAN	N/A				339	Y	Y	1	Y	38 kDa	
Q7Z2K6	Endoplasmic reticulum metallopeptidase 1	ERMP1_HUMAN	Model on 3PB6X	40	Asn182	52.21	904	N	Y	4	Y	100 kDa	
A0FGR8-6	Isoform 6 of Extended synaptotagmin-2	ESYT2_HUMAN	4NPK		Asn433	46.4	921	N	Y	7	P	105 kDa	
			4NPK		Asn511	78.6							
			4NPK		Asn587	52.63							
			4NPK		Asn643	98.77							
Q10471	Polypeptide N-acetylgalactosaminyltransferase 2	GALT2_HUMAN	2FFU		Asn516	139.38	571	Y	Y	1	P	65 kDa	
B7ZAQ6	Golgi pH regulator A	GPHRA_HUMAN	N/A				455	N	Y	3	P	53 kDa	
Q9P035	Very-long-chain (3R)-3-hydroxyacyl-[acyl-carrier protein] dehydratase 3	HACD3_HUMAN	N/A				362	N	Y	3	P	43 kDa	
Q8TCT9	Minor histocompatibility antigen H13	HM13_HUMAN	N/A				377	Y	Y	4	Y	41 kDa	
A1L0T0	Acetolactate synthase-like protein	ILVBL_HUMAN	N/A				632	N	Y	1	P	68 kDa	
Q14573	Inositol 1,4,5-trisphosphate receptor type 3	ITPR3_HUMAN	Model on 3UJ0A	75	Asn104	4.01	2671	N	Y	16	P	304 kDa	
			Model on 3UJ0A		Asn216	111.91							
P13473	Lysosome-associated membrane glycoprotein 2	LAMP2_HUMAN	N/A				410	Y	Y	16	Y	45 kDa	
Q16706	Alpha-mannosidase 2	MA2A1_HUMAN	Model on 3bvxA	41	Asn1125	105.28	1144	N	Y	5	Y	131 kDa	
			Model on 3bvxA		Asn1130	27.58							
Q96N66	Lysophospholipid acyltransferase 7	MBOA7_HUMAN	N/A				472	N	Y	1	P	53 kDa	
Q14728	Major facilitator superfamily domain-containing protein 10	MFS10_HUMAN	N/A				455	N	Y	1	P	48 kDa	
Q6PIU2	Neutral cholesterol ester hydrolase 1	NCEH1_HUMAN	Model on 3ainA	40	Asn270	8.01	408	Y	Y	3	Y	46 kDa	
			Model on 3ainA		Asn287	42.73							
Q969V3	Nicalin	NCLN_HUMAN	N/A				563	Y	Y	2	Y	63 kDa	
Q15738	Sterol-4-alpha-carboxylate 3-dehydrogenase, decarboxylating	NSDHL_HUMAN	N/A				373	N	Y	1	P	42 kDa	
Q8NF37	Lysophosphatidylcholine acyltransferase 1	PCAT1_HUMAN	N/A				534	N	Y	1	P	59 kDa	
Q9UHG3	Prenylcysteine oxidase 1	PCYOX_HUMAN	N/A				505	Y	Y	3	Y	57 kDa	
Q92508	Piezo-type mechanosensitive ion channel component 1	PIEZ1_HUMAN	N/A				2521	Y	Y	8	Y	287 kDa	
Q96S52	GPI transamidase component PIG-S	PIGS_HUMAN	N/A				555	Y	Y	2	Y	62 kDa	
P20340	Cluster of Ras-related protein Rab-6A	RAB6A_HUMAN	1yzq		Asn126	2.09	208	N	Y	1	P	24 kDa	
P04844	Dolichyl-diphosphooligosaccharide--protein glycosyltransferase subunit 2	RPN2_HUMAN	N/A				631	Y	Y	3	Y	69 kDa	
Q6P1M0	Long-chain fatty acid transport protein 4	S27A4_HUMAN	N/A				643	N	Y	4	P	72 kDa	
Q96K37	Solute carrier family 35 member E1	S35E1_HUMAN	N/A				410	N	Y	2	P	45 kDa	
Q8N357	Solute carrier family 35 member F6	S35F6_HUMAN	N/A				371	Y	Y	3	Y	40 kDa	
Q92504	Zinc transporter SLC39A7	S39A7_HUMAN	N/A				469	N	Y	1	P	50 kDa	
O15126	Secretory carrier-associated membrane protein 1	SCAM1_HUMAN	N/A				338	N	Y	2	P	38 kDa	
Q9UGP8	Translocation protein SEC63 homolog	SEC63_HUMAN	N/A				760	N	Y	2	P	88 kDa	
P61009	Signal peptidase complex subunit 3	SPCS3_HUMAN	N/A				180	N	Y	1	Y	20 kDa	
Q9UNL2	Translocon-associated protein subunit gamma	SSRG_HUMAN	N/A				185	N	Y	2	P	21 kDa	
P46977	Dolichyl-diphosphooligosaccharide--protein glycosyltransferase subunit STT3A	STT3A_HUMAN	N/A				705	Y	Y	4	Y	81 kDa	
Q8TCJ2	Dolichyl-diphosphooligosaccharide--protein glycosyltransferase subunit STT3B	STT3B_HUMAN	N/A				826	N	Y	6	P	94 kDa	
Q03518	Antigen peptide transporter 1	TAP1_HUMAN	N/A				808	N	Y	5	P	87 kDa	
Q9NZ01	Very-long-chain enoyl-CoA reductase	TECR_HUMAN	N/A				308	N	Y	3	Y	36 kDa	
O15321	Transmembrane 9 superfamily member 1	TM9S1_HUMAN	N/A				606	N	Y	4	Y	69 kDa	
Q9HD45	Transmembrane 9 superfamily member 3	TM9S3_HUMAN	N/A				589	Y	Y	2	Y	68 kDa	
Q92544	Transmembrane 9 superfamily member 4	TM9S4_HUMAN	N/A				642	Y	Y	1	P	75 kDa	
Q9Y3B3	Transmembrane emp24 domain-containing protein 7	TMED7_HUMAN	N/A				224	Y	Y	1	Y	25 kDa	
Q9BVK6	Transmembrane emp24 domain-containing protein 9	TMED9_HUMAN	N/A				235	N	Y	1	Y	27 kDa	
P49755	Transmembrane emp24 domain-containing protein 10	TMEDA_HUMAN	N/A				219	Y	Y	1	Y	25 kDa	
Q9Y320	Thioredoxin-related transmembrane protein 2	TMX2_HUMAN	N/A				296	Y	Y	2	P	34 kDa	
P02788	Lactotransferrin	TRFL_HUMAN	1LCF		Asn156	129.24	710	Y	N	3	Y	78 kDa	
			1LCF		Asn497	129.34							
			1LCF		Asn642	144.08							
Q9P0L0	Vesicle-associated membrane protein-associated protein A	VAPA_HUMAN	N/A				249	N	Y	4	P	28 kDa	
O95292	Vesicle-associated membrane protein-associated protein B/C	VAPB_HUMAN	N/A				243	N	Y	1	P	27 kDa	
O76024	Wolframin	WFS1_HUMAN	N/A				890	N	Y	5	Y	100 kDa	
					**Mean**	**59.44**							
					**SD**	**46.58**							

MCF7 Cell surface (Unique)

Acc No	Identified Proteins	Uniprot ID	PDB or Model	%matches	Asn Site	Accessibility	Protein Length	Signal Peptide	Transmembrane	ASN SITE (NO)	Glycoprotein	Mol Weight	
Q5TH69	Brefeldin A-inhibited guanine nucleotide-exchange protein 3	BIG3_HUMAN	N/A				2177	N	Y	7	P	241 kDa	N = No
P13987	CD59 glycoprotein	CD59_HUMAN	2J8B		Asn43	90.78	128	Y	Y	1	Y	14 kDa	Y = Yes
P21926	CD9 antigen	CD9_HUMAN	N/A				228	Y	Y	2	Y	25 kDa	P = Potential
Q9HCU4	Cadherin EGF LAG seven-pass G-type receptor 2	CELR2_HUMAN	Model on 1x2mA		Asn81	76.1	2923	Y	Y	19	Y	317 kDa	N/A = Not available
Q8IWA5	Choline transporter-like protein 2	CTL2_HUMAN	N/A				706	Y	Y	4	Y	80 kDa	
P08174	Complement decay-accelerating factor	DAF_HUMAN	1OK3		Asn95	48.58	381	Y	Y	1	Y	41 kDa	
P22413	Ectonucleotide pyrophosphatase/phosphodiesterase family member 1	ENPP1_HUMAN	Model on 4b56	80	Asn179	24.32	925	Y	Y	11	Y	105 kDa	
			Model on 4b56		Asn285	84.77							
			Model on 4b56		Asn341	71.32							
			Model on 4b56		Asn477	70.12							
			Model on 4b56		Asn578	132.45							
			Model on 4b56		Asn585	37.51							
			Model on 4b56		Asn643	113.01							
			Model on 4b56		Asn700	108.15							
			Model on 4b56		Asn731	129.34							
			Model on 4b56		Asn748	149.21							
P29317	Ephrin type-A receptor 2	EPHA2_HUMAN	3C8X		Asn407	108.79	976	Y	Y	2	Y	108 kDa	
			3C8X		Asn435	86.11							
P29323	Ephrin type-B receptor 2	EPHB2_HUMAN	3ZFM		Asn704	53.81	1055	Y	Y	5	Y	117 kDa	
P54753	Ephrin type-B receptor 3	EPHB3_HUMAN	N/A				998	Y	Y	3	Y	110 kDa	
P54760	Ephrin type-B receptor 4	EPHB4_HUMAN	2VWX		Asn768	87.93	987	Y	Y	4	Y	108 kDa	
P37268	Squalene synthase	FDFT_HUMAN	3VJ8		Asn48	56.35	417	N	Y	2	P	48 kDa	
			3VJ8		Asn282	22.26							
Q7L5L3	Glycerophosphodiester phosphodiesterase domain-containing protein 3	GDPD3_HUMAN	N/A				318	N	Y	1	P	37 kDa	
Q08379	Golgin subfamily A member 2	GOGA2_HUMAN	N/A				1002	N	Y	2	P	113 kDa	

P05362	Intercellular adhesion molecule 1	ICAM1_HUMAN	1IAM		Asn130	108.22	532	Y	Y	8	Y	58 kDa
			1IAM		Asn145	58.32						
			1IAM		Asn183	86.77						
			1IAM		Asn202	51.58						
			2OZ4		Asn267	145.78						
			2OZ4		Asn296	163.34						
			2OZ4		Asn385	102.35						
			2OZ4		Asn406	63.14						
P08069	Insulin-like growth factor 1 receptor	IGF1R_HUMAN	2IGR		Asn51	76.06	1367	Y	Y	17	Y	155 kDa
			2IGR		Asn102	44.86						
			2IGR		Asn135	35.48						
			2IGR		Asn244	60.52						
			2IGR		Asn314	92.37						
			2IGR		Asn417	99.23						
			2IGR		Asn438	99.42						
O75054	Immunoglobulin superfamily member 3	IGSF3_HUMAN	N/A				1194	Y	Y	12	Y	135 kDa
P17301	Integrin alpha-2	ITA2_HUMAN	1DZI		Asn343	52.82	1181	Y	Y	10	Y	129 kDa
P26006	Integrin alpha-3	ITA3_HUMAN	N/A				1051	Y	Y	14	Y	117 kDa
P05556	Integrin beta-1	ITB1_HUMAN	3VI3		Asn94	103.93	798	Y	Y	12	Y	88 kDa
			3VI3		Asn97	67.29						
			3VI3		Asn212	114.74						
			3VI3		Asn269	114.84						
			3VI3		Asn363	73.91						
			3VI3		Asn406	86.16						
			3VI3		Asn417	109.5						
P16144	Integrin beta-4	ITB4_HUMAN	N/A				1822	Y	Y	8	Y	202 kDa
P18084	Integrin beta-5	ITB5_HUMAN	Model on 4g1e	56	Asn347	95.88	799	Y	Y	10	Y	88 kDa
			Model on 4g1e		Asn460	54.83						
			Model on 4g1e		Asn477	89.39						
			Model on 4g1e		Asn552	59.16						
			Model on 4g1e		Asn586	84.71						
			Model on 4g1e		Asn654	100.92						
			Model on 4g1e		Asn705	25.07						
P18564	Integrin beta-6	ITB6_HUMAN	Model on 3FCS	53	Asn48	95.63	788	Y	Y	10	Y	86 kDa
			Model on 3FCS		Asn97	2.71						
			Model on 3FCS		Asn260	135.49						
			Model on 3FCS		Asn387	30.46						
			Model on 3FCS		Asn396	142.45						
			Model on 3FCS		Asn463	99.28						
			Model on 3FCS		Asn471	31.78						
			Model on 3FCS		Asn541	61.38						
			Model on 3FCS		Asn575	59.2						
Q9H0X4	Protein ITFG3	ITFG3_HUMAN	N/A				552	N	Y	8	Y	60 kDa
P15529-2	Isoform B of Membrane cofactor protein	MCP_HUMAN	3O8E		Asn83	130.71	392	Y	Y	3	Y	44 kDa
			3O8E		Asn114	53.41						
			3O8E		Asn273	110.84						
P11717	Cation-independent mannose-6-phosphate receptor	MPRI_HUMAN	2CBZ		Asn648	89.3	2491	Y	Y	21	Y	274 kDa
			2CBZ		Asn719	97.06						
			2CBZ		Asn767	140.84						
			2CBZ		Asn819	119.92						
O95297	Myelin protein zero-like protein 1	MPZL1_HUMAN	Model on 1neuA		Asn50	66.93	269	Y	Y	3	Y	29 kDa
			Model on 1neuA	43	Asn131	15.78						
Q04721	Neurogenic locus notch homolog protein 2	NOTC2_HUMAN	2F8Y		Asn1922	89.22	2471	Y	Y	9	Y	265 kDa
Q9UM47	Neurogenic locus notch homolog protein 3	NOTC3_HUMAN	Model on 2f8yA	83	Asn2066	47.74	2321	Y	Y	4	Y	244 kDa
Q9Y4D7	Plexin-D1	PLXD1_HUMAN	Model on 3ig3A	49	Asn1341	0.14	1925	Y	Y	20	Y	212 kDa
			Model on 3ig3A		Asn1559	48.75						
Q15437	Protein transport protein Sec23B	SC23B_HUMAN	Model on 2nupA	86	Asn241	60.29	767	N	Y	5	P	86 kDa
			Model on 2nupA		Asn338	68.48						
			Model on 2nupA		Asn367	30.64						
			Model on 2nupA		Asn380	43.48						
			Model on 2nupA		Asn596	146.73						
P53992	Protein transport protein Sec24C	SC24C_HUMAN	3EH2		Asn368	9.91	1094	N	Y	5	P	118 kDa
			3EH2		Asn592	71						
			3EH2		Asn774	67.42						
Q8WTV0-4	Isoform 4 of Scavenger receptor class B member 1	SCRB1_HUMAN	N/A				552	Y	Y	9	Y	54 kDa
O15270	Serine palmitoyltransferase 2	SPTC2_HUMAN	N/A				562	N	Y	1	P	63 kDa
Q9Y5Y6	Suppressor of tumorigenicity 14 protein	ST14_HUMAN	3NCL		Asn772	73.15	855	N	Y	4	Y	95 kDa
Q6NUQ4	Transmembrane protein 214	TM214_HUMAN	N/A				689	N	Y	4	Y	77 kDa
Q9H1E5	Thioredoxin-related transmembrane protein 4	TMX4_HUMAN	Model on 1x5eA	50	Asn46	47.43	349	Y	Y	1	P	39 kDa
Q8NFA0	Ubiquitin carboxyl-terminal hydrolase 32	UBP32_HUMAN	Model on 2y6eA	58	Asn746	17.61	1604	N	Y	13	P	182 kDa
			Model on 2y6eA		Asn772	9.21						
			Model on 2y6eA		Asn875	15.3						
					**Mean**	**76.20**						
					**SD**	**38.13**						

MCF7 secreted (Unique)

Acc No	Identified Proteins	Uniprot ID	PDB or Model	%matches	Asn Site	Accessibility	Protein Length	Signal Peptide	Transmembrane	ASN SITE (NO)	Glycoprotein	Mol Weight
P05067	Amyloid beta A4 protein	A4_HUMAN	3UMH		Asn467	74.25	770	Y	Y	2	Y	87 kDa
P01011	Alpha-1-antichymotrypsin	AACT_HUMAN	1AS4		Asn93	112.72	423	Y	N	6	Y	48 kDa
			1AS4		Asn106	88.7						
			1AS4		Asn127	76.52						
			1AS4		Asn186	123.36						
			1AS4		Asn271	86.61						
O14672	Disintegrin and metalloproteinase domain-containing protein 10	ADA10_HUMAN	Model on 2ao7A	92	Asn551	71.72	748	Y	Y	4	Y	84 kDa
Q06481	Amyloid-like protein 2	APLP2_HUMAN	Model on 3umh	69	Asn541	79.78	763	Y	Y	1	Y	87 kDa
P15291	Beta-1,4-galactosyltransferase 1	B4GT1_HUMAN	N/A				398	N	Y	1	Y	44 kDa
P18075	Bone morphogenetic protein 7	BMP7_HUMAN	1LXI		Asn372	65.32	431	Y	N	4	Y	49 kDa
P43251	Biotinidase	BTD_HUMAN	N/A				543	Y	N	6	Y	61 kDa
P09668	Pro-cathepsin H	CATH_HUMAN	Model on 8pchA	92	Asn230	98.87	335	Y	N	3	Y	37 kDa
P16870	Carboxypeptidase E	CBPE_HUMAN	Model on 2nsmA	54	Asn139	62.26	476	Y	N	2	Y	53 kDa
			Model on 2nsmA		Asn390	98.55						
Q6YHK3	CD109 antigen	CD109_HUMAN	N/A				1445	Y	Y	19	Y	162 kDa
P10909	Clusterin	CLUS_HUMAN	N/A				449	Y	N	7	Y	52 kDa
P20908	Collagen alpha-1(V) chain	CO5A1_HUMAN	N/A				1838	Y	N	5	Y	184 kDa
Q99715	Collagen alpha-1(XII) chain	COCA1_HUMAN	N/A				3063	Y	N	9	Y	333 kDa
P39060	Collagen alpha-1(XVIII) chain	COIA1_HUMAN	N/A				1754	Y	N	5	Y	178 kDa
O94985-2	Isoform 2 of Calsyntenin-1	CSTN1_HUMAN	N/A				981	Y	Y	4	Y	109 kDa
Q9H4D0	Calsyntenin-2	CSTN2_HUMAN	N/A				955	Y	Y	6	Y	107 kDa
Q9BQT9	Calsyntenin-3	CSTN3_HUMAN	N/A				956	Y	Y	5	Y	106 kDa
Q14118	Dystroglycan	DAG1_HUMAN	Model on 1u2cA	95	Asn141	114.54	895	Y	Y	6	Y	97 kDa
			1EG4		Asn882	196.79						
O94907	Dickkopf-related protein 1	DKK1_HUMAN	N/A				266	Y	N	1	Y	29 kDa
P30040	Endoplasmic reticulum resident protein 29	ERP29_HUMAN	2QC7		Asn187	28.6	261	Y	N	1	Y	29 kDa
P02765	Alpha-2-HS-glycoprotein	FETUA_HUMAN	N/A				367	Y	N	2	Y	39 kDa
P02751	Fibronectin	FINC_HUMAN	3M7P		Asn430	74.08	2386	Y	N	11	Y	263 kDa
			3M7P		Asn528	83.89						
			3M7P		Asn542	42.9						
			1FNFN		Asn1236	80.19						
			1FNFN		Asn1244	48.71						
			1FNFN		Asn1417	140.61						
			3R8Q		Asn1813	61.87						
Q9BTY2	Plasma alpha-L-fucosidase	FUCO2_HUMAN	N/A				467	Y	N	4	Y	54 kDa
Q99988	Growth/differentiation factor 15	GDF15_HUMAN	N/A				308	Y	N	2	Y	34 kDa
Q92820	Gamma-glutamyl hydrolase	GGH_HUMAN	1L9X		Asn116	70.56	318	Y	N	4	Y	36 kDa
			1L9X		Asn163	66.09						
			1L9X		Asn203	43.44						
			1L9X		Asn307	93.36						
P35052	Glypican-1	GPC1_HUMAN	4ACR		Asn79	103.6	558	Y	Y	2	Y	62 kDa
			4ACR		Asn116	73.03						
O75487	Glypican-4	GPC4_HUMAN	Model on 4acrB	47	Asn268	31.08	556	Y	Y	1	Y	62 kDa
			Model on 4acrB		Asn406	78.51						
P05154	Plasma serine protease inhibitor	IPSP_HUMAN	3DY0		Asn248	118.66	406	Y	N	3	Y	46 kDa
			3DY0		Asn261	107.12						
			3DY0		Asn338	111.47						
P55268	Laminin subunit beta-2	LAMB2_HUMAN	Model on 4aqsA	68	Asn248	23.47	1798	Y	N	7	Y	196 kDa
			Model on 4aqsA		Asn368	86.45						
P11047	Laminin subunit gamma-1	LAMC1_HUMAN	Model on 4aqtA	97	Asn60	115.11	1609	Y	N	14	Y	178 kDa
			Model on 4aqtA		Asn134	45.97						
Q08380	Galectin-3-binding protein	LG3BP_HUMAN	1BY2		Asn69	69.64	585	Y	N	7	Y	65 kDa
			1BY2		Asn125	131.42						
Q14766	Latent-transforming growth factor beta-binding protein 1	LTBP1_HUMAN	Model on 1lmjA	50	Asn1197	120.57	1721	Y	N	8	Y	187 kDa
			Model on 1lmjA	99	Asn1366	87.73						

N = No
Y = Yes
P = Potential
N/A = Not available

P10253	Lysosomal alpha-glucosidase	LYAG_HUMAN	Model on 3l4ya	44	Asn140	125.92	952	N	Y	7	Y	105 kDa
			Model on 3l4ya		Asn470	34.89						
			Model on 3l4ya		Asn652	69.47						
			Model on 3l4ya		Asn882	75.95						
			Model on 3l4ya		Asn925	91.02						
O95274	Ly6/PLAUR domain-containing protein 3	LYPD3_HUMAN	N/A				346	Y	Y	6	Y	36 kDa
P33908	Mannosyl-oligosaccharide 1,2-alpha-mannosidase IA	MA1A1_HUMAN	Model on 1nxca	92	Asn513	71.29	653	Y	Y	1		73 kDa
P69849	Nodal modulator 3	NOMO3_HUMAN	N/A				1222	Y	Y	7	Y	134 kDa
Q6UXI9	Nephronectin	NPNT_HUMAN	N/A				565	Y	N	1	Y	62 kDa
P98160	Basement membrane-specific heparan sulfate proteoglycan core protein	PGBM_HUMAN	N/A				4391	Y	N	10	Y	469 kDa
O60568	Procollagen-lysine,2-oxoglutarate 5-dioxygenase 3	PLOD3_HUMAN	N/A				738	Y	N	2	Y	85 kDa
P23470	Receptor-type tyrosine-protein phosphatase gamma	PTPRG_HUMAN	3JXH		Asn109	92.44	1445	Y	Y	13		162 kDa
			3JXH		Asn113	90.11						
			3JXH		Asn156	113.58						
			2NLK		Asn863	119.8						
			2NLK		Asn1181	49.39						
Q92626	Peroxidasin homolog	PXDN_HUMAN	N/A				1479	Y	N	11	Y	165 kDa
O00391	Sulfhydryl oxidase 1	QSOX1_HUMAN	3Q6O		Asn130	149.4	747	Y	Y	4	Y	83 kDa
			3Q6O		Asn243	125.74						
Q9Y6N7	Roundabout homolog 1	ROBO1_HUMAN	4HLJ		Asn820	103.57	1651	Y	Y	15	Y	181 kDa
			4HLJ		Asn827	96.61						
Q6UXD5-3	Isoform 3 of Seizure 6-like protein 2	SE6L2_HUMAN	N/A				910	Y	Y	9	Y	92 kDa
Q99985	Semaphorin-3C	SEM3C_HUMAN	Model on 1q47A	51	Asn81	10.15	751	Y	N	7	Y	85 kDa
			Model on 1q47A		Asn123	62.24						
			Model on 1q47A		Asn252	154.01						
			Model on 1q47A		Asn268	40.77						
			Model on 1q47A		Asn465	112.35						
Q13275	Cluster of Semaphorin-3F	SEM3F_HUMAN	Model on 1q47A	54	Asn53	85.05	785	Y	N	2	Y	88 kDa
			Model on 1q47A		Asn126	28.49						
Q9HAT2	Sialate O-acetylesterase	SIAE_HUMAN	N/A				523	Y	N	6	Y	58 kDa
Q92673	Sortilin-related receptor	SORL_HUMAN	Model on 1j8e	60	Asn1164	81.91	2214	Y	Y	27	Y	248 kDa
			Model on 1j8e		Asn1191	60.75						
			Model on 2dm4A	92	Asn1706	46.31						
Q99523	Sortilin	SORT_HUMAN	3F6K		Asn98	134.06	831	Y	Y	7	Y	92 kDa
			3F6K		Asn162	99.51						
			3F6K		Asn274	139.63						
			3F6K		Asn406	68.9						
			3F6K		Asn582	19.36						
			3F6K		Asn684	127.41						
P52823	Stanniocalcin-1	STC1_HUMAN	N/A				247	Y	N	2	Y	28 kDa
O76061	Stanniocalcin-2	STC2_HUMAN	N/A				302	Y	N	1	Y	33 kDa
Q8IWU5-2	Isoform 2 of Extracellular sulfatase Sulf-2	SULF2_HUMAN	N/A				870	Y	Y	12	Y	100 kDa
Q24JP5	Transmembrane protein 132A	T132A_HUMAN	N/A				1023	Y	Y	3	Y	110 kDa
P01033	Metalloproteinase inhibitor 1	TIMP1_HUMAN	3V96		Asn101	83.1	207	Y	N	2	Y	23 kDa
					**Mean**	**85.63**						
					**SD**	**35.47**						

MDA468 Microsome (Unique)

Acc No	Identified Proteins	Uniprot ID	PDB or Model	%matches	Asn Site	Accessibility	Protein Length	Signal Peptide	Trans-membrane	ASN SITE (NO)	Glycoprotein	Mol Weight
P28288	ATP-binding cassette sub-family D member 3	ABCD3_HUMAN	N/A				659	Y	Y	1	N	75 kDa
O00767	Acyl-CoA desaturase	ACOD_HUMAN	N/A				359	Y	P	3	N	42 kDa
P15144	Aminopeptidase N	AMPN_HUMAN	N/A				967	Y	Y	11	Y	110 kDa
Q9HD20	Probable cation-transporting ATPase 13A1	AT131_HUMAN	N/A				1204	Y	Y	6	N	133 kDa
P16615	Sarcoplasmic/endoplasmic reticulum calcium ATPase 2	AT2A2_HUMAN	Model on 3ar4	84	Asn19	78.53	1042	Y	P	7	N	115 kDa
			Model on 3ar4		Asn421	10.53						
			Model on 3ar4		Asn589	71.33						
			Model on 3ar4		Asn738	6.43						
			Model on 3ar4		Asn918	46.93						
			Model on 3ar4		Asn962	75.61						
P23634	Cluster of Plasma membrane calcium-transporting ATPase 4	AT2B4_HUMAN	N/A				1241	Y	P	3	N	138 kDa
Q8NHH9	Atlastin-2	ATLA2_HUMAN	Model on 4IDO	73	Asn163	116.43	583	Y	P	2	N	66 kDa
			Model on 4IDO		Asn204	1.64						
Q6DD88	Atlastin-3	ATLA3_HUMAN	Model on 3q5E	69	Asn173	2.3	541	Y	P	5	N	61 kDa
			Model on 3q5E		Asn312	11.41						
P35613-2	Isoform 2 of Basigin	BASI_HUMAN	3I84		Asn160	82.87	385	N	Y	5	Y	29 kDa
Q9NR09	Baculoviral IAP repeat-containing protein 6	BIRC6_HUMAN	N/A				4857	Y	Y	28	Y	530 kDa
P27824	Calnexin	CALX_HUMAN	N/A				592	Y	Y	1	Y	68 kDa
Q96A33	Coiled-coil domain-containing protein 47	CCD47_HUMAN	N/A				483	Y	Y	1	Y	56 kDa
Q96S66	Chloride channel CLIC-like protein 1	CLCC1_HUMAN	N/A				551	Y	Y	1	Y	62 kDa
O96005	Cleft lip and palate transmembrane protein 1	CLPT1_HUMAN	N/A				669	Y	Y	6	N	76 kDa
Q16850	Lanosterol 14-alpha demethylase	CP51A_HUMAN	3ld6		Asn483	32.36	503	Y	Y	1	Y	57 kDa
Q9UBM7	7-dehydrocholesterol reductase	DHCR7_HUMAN	N/A				475	Y	P	2	N	54 kDa
Q9BW60	Elongation of very long chain fatty acids protein 1	ELOV1_HUMAN	N/A				279	Y	P	2	N	33 kDa
Q9NYP7	Elongation of very long chain fatty acids protein 5	ELOV5_HUMAN	N/A				299	Y	P	1	N	35 kDa
Q8N766	ER membrane protein complex subunit 1	EMC1_HUMAN	N/A				993	Y	Y	3	Y	112 kDa
P33947	Cluster of ER lumen protein retaining receptor 2	ERD22_HUMAN	N/A				212	Y	P	1	N	24 kDa
Q9UKR5	Probable ergosterol biosynthetic protein 28	ERG28_HUMAN	N/A				140	Y	P	1	N	16 kDa
Q7Z2K6	Endoplasmic reticulum metallopeptidase 1	ERMP1_HUMAN	Model on 3PB6X	40	Asn182	52.21	904	Y	Y	4	N	100 kDa
Q9BSJ8	Extended synaptotagmin-1	ESYT1_HUMAN	Model on 2dmga	46	Asn1062	54.85	1104	Y	P	3	N	123 kDa
A0FGR8-6	Isoform 6 of Extended synaptotagmin-2	ESYT2_HUMAN	N/A				921	Y	P	7	N	105 kDa
P37268	Squalene synthase	FDFT_HUMAN	3VJ8		Asn48	56.35	417	Y	P	2	N	48 kDa
			3VJ8		Asn282	22.26						
Q10471	Polypeptide N-acetylgalactosaminyltransferase 2	GALT2_HUMAN	2FFU		Asn516	139.38	571	Y	P	1	Y	65 kDa
P14314	Glucosidase 2 subunit beta	GLU2B_HUMAN	N/A				528	N	Y	2	Y	59 kDa
Q92896	Golgi apparatus protein 1	GSLG1_HUMAN	N/A				1179	Y	Y	5	Y	135 kDa
Q8TCT9	Minor histocompatibility antigen H13	HM13_HUMAN	N/A				377	Y	Y	4	Y	41 kDa
P07099	Epoxide hydrolase 1	HYEP_HUMAN	N/A				455	Y	P	2	Y	53 kDa
P23229-2	Isoform Alpha-6X1A of Integrin alpha-6	ITA6_HUMAN	N/A				1130	Y	Y	10	Y	119 kDa
Q14571	Inositol 1,4,5-trisphosphate receptor type 2	ITPR2_HUMAN	Model on 3jrra	82	Asn82	154.25	2701	Y	P	18	N	308 kDa
			Model on 3jrra		Asn103	10.53						
Q14573	Inositol 1,4,5-trisphosphate receptor type 3	ITPR3_HUMAN	Model on 3jrra	82	Asn215	133.79	2671	Y	P	16	N	304 kDa
			Model on 3uj0a	75	Asn104	4.01						
			Model on 3uj0a		Asn216	111.91						
Q92604	Acyl-CoA:lysophosphatidylglycerol acyltransferase 1	LGAT1_HUMAN	N/A				370	Y	P	2	N	43 kDa
Q96N66	Lysophospholipid acyltransferase 7	MBOA7_HUMAN	N/A				472	Y	P	1	N	53 kDa
Q13724	Mannosyl-oligosaccharide glucosidase	MOGS_HUMAN	N/A				837	Y	Y	2	Y	92 kDa
Q6PIU2	Neutral cholesterol ester hydrolase 1	NCEH1_HUMAN	Model on 3ainA	40	Asn270	8.01	408	Y	Y	3	Y	46 kDa
			Model on 3ainA		Asn287	42.73						
Q969V3	Nicalin	NCLN_HUMAN	N/A				563	Y	Y	2	Y	63 kDa
P08473	Neprilysin	NEP_HUMAN	1r1H		Asn145	64.42	750	Y	Y	6	Y	86 kDa
			1r1H		Asn285	113.85						
			1r1H		Asn311	96.09						
			1r1H		Asn325	57.51						
			1r1H		Asn335	147.36						
			1r1H		Asn628	106.17						
Q15738	Sterol-4-alpha-carboxylate 3-dehydrogenase, decarboxylating	NSDHL_HUMAN	N/A				373	Y	P	1	N	42 kDa
Q9UHG3	Prenylcysteine oxidase 1	PCYOX_HUMAN	N/A				505	Y	Y	3	Y	57 kDa
Q8TEQ8	GPI ethanolamine phosphate transferase 3	PIGO_HUMAN	N/A				1089	Y	Y	2	Y	119 kDa
Q8IY17	Neuropathy target esterase	PLPL6_HUMAN	N/A				1366	Y	Y	4	Y	150 kDa
P05187	Alkaline phosphatase, placental type	PPB1_HUMAN	1ZED		Asn144	118.69	535	N	Y	2	Y	58 kDa
					Asn271	19.37						
P20340	Cluster of Ras-related protein Rab-6A	RAB6A_HUMAN	1YZQ		Asn126	2.09	208	Y	P	1	N	24 kDa
Q8TC12	Retinol dehydrogenase 11	RDH11_HUMAN	N/A				318	Y	P	2	Y	35 kDa
Q6NUM9	All-trans-retinol 13,14-reductase	RETST_HUMAN	N/A				610	Y	Y	1	Y	67 kDa
P04843	Dolichyl-diphosphooligosaccharide--protein glycosyltransferase subunit 1	RPN1_HUMAN	N/A				607	Y	Y	2	Y	69 kDa
P04844	Dolichyl-diphosphooligosaccharide--protein glycosyltransferase subunit 2	RPN2_HUMAN	N/A				631	Y	Y	3	Y	69 kDa
Q9P2E9	Ribosome-binding protein 1	RRBP1_HUMAN	N/A				1410	Y	P	3	N	152 kDa
Q9NQC3-2	Isoform 2 of Reticulon-4	RTN4_HUMAN	N/A				1192	Y	P	7	N	40 kDa
Q9BXP2	Solute carrier family 12 member 9	S12A9_HUMAN	N/A				914	Y	Y	3	N	96 kDa
Q92504	Zinc transporter SLC39A7	S39A7_HUMAN	N/A				469	Y	P	1	N	50 kDa
P43007	Neutral amino acid transporter A	SATT_HUMAN	N/A				532	Y	Y	3	Y	56 kDa
O75396	Vesicle-trafficking protein SEC22b	SC22B_HUMAN	N/A				215	Y	P	1	N	25 kDa
O15126	Secretory carrier-associated membrane protein 1	SCAM1_HUMAN	N/A				338	Y	P	2	N	38 kDa
Q9UGP8	Translocation protein SEC63 homolog	SEC63_HUMAN	N/A				760	Y	P	2	N	88 kDa
P50454	Serpin H1	SERPH_HUMAN	Model on 3zhaA	99	Asn120	113.05	418	N	Y	2	Y	46 kDa
			Model on 3zhaA		Asn125	64.4						
O43291	Kunitz-type protease inhibitor 2	SPIT2_HUMAN	Model on 4isoB	59	Asn57	31.32	252	Y	Y	2	Y	28 kDa
P43307	Translocon-associated protein subunit alpha	SSRA_HUMAN	N/A				286	Y	Y	2	Y	32 kDa
Q9UNL2	Translocon-associated protein subunit gamma	SSRG_HUMAN	N/A				185	Y	P	2	Y	21 kDa

N = No
Y = Yes
P = Potential
N/A = Not available

P46977	Dolichyl-diphosphooligosaccharide--protein glycosyltransferase subunit STT3A	STT3A_HUMAN	N/A				705	Y	Y	4	Y	81 kDa
Q8TCJ2	Dolichyl-diphosphooligosaccharide--protein glycosyltransferase subunit STT3B	STT3B_HUMAN	N/A				826	Y	P	6	N	94 kDa
Q8NBJ7	Sulfatase-modifying factor 2	SUMF2_HUMAN	1Y4J		Asn191	82.39	301	N	Y	1	Y	34 kDa
Q16563	Synaptophysin-like protein 1	SYPL1_HUMAN	N/A				259	Y	Y	3	N	29 kDa
Q9NX61	Transmembrane protein 161A	T161A_HUMAN	N/A				479	Y	Y	2	Y	54 kDa
Q03518	Antigen peptide transporter 1	TAP1_HUMAN	N/A				808	Y	P	5	N	87 kDa
Q9NZ01	Very-long-chain enoyl-CoA reductase	TECR_HUMAN	N/A				308	Y	Y	3	N	36 kDa
Q92544	Transmembrane 9 superfamily member 4	TM9S4_HUMAN	N/A				642	Y	P	1	Y	75 kDa
P49755	Transmembrane emp24 domain-containing protein 10	TMEDA_HUMAN	N/A				219	Y	Y	1	Y	25 kDa
Q9Y320	Thioredoxin-related transmembrane protein 2	TMX2_HUMAN	Model on 2dj0A	99	Asn165	132.71	296	Y	P	2	Y	34 kDa
			Model on 2dj0A		Asn185	29.89						
O95292	Vesicle-associated membrane protein-associated protein B/C	VAPB_HUMAN	3IKK		Asn68	93.09	243	Y	P	1	N	27 kDa
P38435	Vitamin K-dependent gamma-carboxylase	VKGC_HUMAN	N/A				758	Y	Y	8	N	88 kDa
					**Mean**	**64.98**						
					**SD**	**46.99**						

MDA468 Cell surface (Unique)

Acc No	Identified Proteins	Uniprot ID	PDB or Model	%matches	Asn Site	Accessibility	Protein Length	Signal Peptide	Transmembrane	ASN SITE (NO)	Glycoprotein	Mol Weight
P21589	5'-nucleotidase	5NTD_HUMAN	4H2G		Asn53	72.34	574	Y	Y	4	Y	63 kDa
					Asn311	41.82						
					Asn333	92.24						
					Asn403	128.19						
Q92485	Acid sphingomyelinase-like phosphodiesterase 3b	ASM3B_HUMAN	N/A				455	Y	N	3	Y	51 kDa
P50895	Basal cell adhesion molecule	BCAM_HUMAN	N/A				628	Y	Y	5	Y	67 kDa
P12830	Cadherin-1	CADH1_HUMAN	N/A				882	Y	Y	6	Y	97 kDa
P08571	Monocyte differentiation antigen CD14	CD14_HUMAN	4GLP		Asn37	77.49	375	Y	N	4	Y	40 kDa
					Asn151	61.64						
					Asn282	35.57						
					Asn323	73.13						
Q9H5V8	CUB domain-containing protein 1	CDCP1_HUMAN	N/A				836	Y	Y	15	Y	93 kDa
P14625	Endoplasmin	ENPL_HUMAN	4NH9		Asn107	51.8	803	Y	N	6	Y	92 kDa
			4NH9		Asn217	151.6						
P54753	Cluster of Ephrin type-B receptor 3	EPHB3_HUMAN	Model on 2X10	40	Asn351	119.4	998	Y	Y	3	Y	110 kDa
			Model on 2X10		Asn445	80.74						
P05362	Intercellular adhesion molecule 1	ICAM1_HUMAN	1IAM		Asn130	108.22	532	Y	Y	8	Y	58 kDa
			1IAM		Asn145	58.32						
			1IAM		Asn183	86.77						
			1IAM		Asn202	51.58						
			2OZ4		Asn267	145.78						
			2OZ4		Asn296	163.34						
			2OZ4		Asn385	102.35						
			2OZ4		Asn406	63.14						
P05556	Integrin beta-1	ITB1_HUMAN	3VI3		Asn94	103.93	798	Y	Y	12	Y	88 kDa
			3VI3		Asn97	67.29						
			3VI3		Asn212	114.74						
			3VI3		Asn269	114.84						
			3VI3		Asn363	73.91						
			3VI3		Asn406	86.16						
			3VI3		Asn417	109.5						
P18084	Integrin beta-5	ITB5_HUMAN	Model on 4g1e	56	Asn347	95.88	799	Y	Y	10	Y	88 kDa
			Model on 4g1e		Asn460	54.83						
			Model on 4g1e		Asn477	89.39						
			Model on 4g1e		Asn552	59.16						
			Model on 4g1e		Asn586	84.71						
			Model on 4g1e		Asn654	100.92						
			Model on 4g1e		Asn705	25.07						
Q9Y624	Junctional adhesion molecule A	JAM1_HUMAN	1NBQ		Asn185	114.68	299	Y	Y	1	Y	33 kDa
P15529	Cluster of Membrane cofactor protein	MCP_HUMAN	3O8E		Asn83	130.71	392	Y	Y	3	Y	44 kDa
			3O8E		Asn114	53.41						
			3O8E		Asn273	110.84						
O95297	Myelin protein zero-like protein 1	MPZL1_HUMAN	Model on 1neuA	43	Asn50	66.93	269	Y	Y	3	Y	29 kDa
			Model on 1neuA		Asn131	15.78						
Q7L5N7	Lysophosphatidylcholine acyltransferase 2	PCAT2_HUMAN	N/A				544	N	Y	1	P	60 kDa
					**Mean**	**85.95**						
					**SD**	**34.08**						

N = No
Y = Yes
P = Potential
N/A = Not available

MDA468 secreted (Unique)

Acc No	Identified Proteins	Uniprot ID	PDB or Model	%matches	Asn Site	Accessibility	Protein Length	Signal Peptide	Transmembrane	ASN SITE (NO)	Glycoprotein	Mol Weight
P01023	Cluster of Alpha-2-macroglobulin	A2MG_HUMAN	4ACQ		Asn55	146.06	1474	Y	N	8	Y	163 kDa
			4ACQ		Asn70	95.14						
			4ACQ		Asn247	96.85						
			4ACQ		Asn396	108.31						
			4ACQ		Asn410	91.42						
			4ACQ		Asn869	101.89						
			4ACQ		Asn991	72.29						
			4ACQ		Asn1424	114.32						
P05067	Amyloid beta A4 protein	A4_HUMAN	3UMH		Asn467	74.25	770	Y	Y	2	Y	87 kDa
O14672	Disintegrin and metalloproteinase domain-containing protein 10	ADA10_HUMAN	Model on 2ao7A	92	Asn551	71.72	748	Y	Y	4	Y	84 kDa
O00468-6	Isoform 6 of Agrin	AGRIN_HUMAN	Model on 1JB3	90	Asn135	54.9	2067	Y	Y	4	Y	215 kDa
Q06481	Amyloid-like protein 2	APLP2_HUMAN	Model on 3umh	69	Asn541	79.78	763	Y	Y	1	Y	87 kDa
P20933	N(4)-(beta-N-acetylglucosaminyl)-L-asparaginase	ASPG_HUMAN	1APY		Asn38	75.77	346	Y	N	2	Y	37 kDa
			1APY		Asn308	29.62						
Q9Y2A9	UDP-GlcNAc:betaGal beta-1,3-N-acetylglucosaminyltransferase 3	B3GN3_HUMAN	N/A				372	Y	N	6	Y	43 kDa
P13497	Bone morphogenetic protein 1	BMP1_HUMAN	3EDH		Asn91	109.12	986	Y	N	5	Y	111 kDa
P53634	Dipeptidyl peptidase 1	CATC_HUMAN	3PDF		Asn29	101.28	463	Y	N	4	P	52 kDa
			3PDF		Asn53	87.11						
			3PDF		Asn119	85.83						
			3PDF		Asn276	112.51						
P09668	Pro-cathepsin H	CATH_HUMAN	Model on 8pcha	92	Asn230	98.87	335	Y	N	3	Y	37 kDa
P00450	Ceruloplasmin	CERU_HUMAN	4ENZ		Asn138	141.96	1065	Y	N	7	Y	122 kDa
			4ENZ		Asn227	26.13						
			4ENZ		Asn358	117.74						
			4ENZ		Asn397	92.06						
			4ENZ		Asn588	26.12						
			4ENZ		Asn762	157.07						
			4ENZ		Asn926	31.51						
P10909	Clusterin	CLUS_HUMAN	N/A				449	Y	N	7	Y	52 kDa
P01024	Complement C3	CO3_HUMAN	2A74		Asn85	69.77	1663	Y	N	3	Y	187 kDa
			2WII		Asn939	106.85						
			2A74		Asn1617	119.8						
P0C0L4	Cluster of Complement C4-A	CO4A_HUMAN	N/A				1744	Y	N	1	Y	193 kDa
Q99715	Collagen alpha-1(XII) chain	COCA1_HUMAN	N/A				3063	Y	N	9	Y	333 kDa
Q9H3G5	Probable serine carboxypeptidase CPVL	CPVL_HUMAN	N/A				476	Y	N	4	Y	54 kDa
O94985	Calsyntenin-1	CSTN1_HUMAN	N/A				981	Y	Y	4	Y	110 kDa
P29279	Connective tissue growth factor	CTGF_HUMAN	N/A				349	Y	N	2	Y	38 kDa
Q08345	Epithelial discoidin domain-containing receptor 1	DDR1_HUMAN	4ag4		Asn211	52.72	913	Y	Y	7	Y	101 kDa
			4ag4		Asn260	52.09						
			4bkj		Asn777	134.16						
O00115	Deoxyribonuclease-2-alpha	DNS2A_HUMAN	N/A				360	Y	N	4	Y	40 kDa
Q9NZ08	Endoplasmic reticulum aminopeptidase 1	ERAP1_HUMAN	2YD0		Asn70	39.31	941	Y	Y	5	Y	107 kDa
			2YD0		Asn154	116.56						
			2YD0		Asn414	81.81						
			2YD0		Asn760	134.76						
			2YD0		Asn901	53.45						
			3AHQ		Asn280	30.3						
Q14517	Protocadherin Fat 1	FAT1_HUMAN	N/A				4588	Y	Y	29	Y	506 kDa
Q9NYQ8	Protocadherin Fat 2	FAT2_HUMAN	N/A				4349	Y	Y	41	Y	479 kDa
P23142	Cluster of Fibulin-1	FBLN1_HUMAN	N/A				703	Y	N	3	Y	77 kDa
Q12805	EGF-containing fibulin-like extracellular matrix protein 1	FBLN3_HUMAN	N/A				493	Y	N	1	Y	55 kDa
P02765	Alpha-2-HS-glycoprotein	FETUA_HUMAN	N/A				367	Y	N	2	Y	39 kDa
P02751	Fibronectin	FINC_HUMAN	3M7P		Asn430	74.08	2386	Y	N	11	Y	263 kDa
			3M7P		Asn528	83.89						
			3M7P		Asn542	42.9						
			1FNFN		Asn1236	80.19						
			1FNFN		Asn1244	48.71						
			1FNFN		Asn1417	140.61						
			3R8Q		Asn1813	61.87						
Q86XX4-2	Isoform 2 of Extracellular matrix protein FRAS1	FRAS1_HUMAN	N/A				4008	Y	Y	17	Y	444 kDa
Q92820	Gamma-glutamyl hydrolase	GGH_HUMAN	1L9X		Asn116	70.56	318	Y	N	4	Y	36 kDa

N = No
Y = Yes
P = Potential
N/A = Not available

			1L9X		Asn163	66.09						
			1L9X		Asn203	43.44					
			1L9X		Asn307	93.36					
P35052	Glypican-1	GPC1_HUMAN	4ACR		Asn79	103.6	558	Y	Y	2	Y	62 kDa
			4ACR		Asn116	73.03					
P06865	Beta-hexosaminidase subunit alpha	HEXA_HUMAN	2GJX		Asn115	62.89	529	Y	Y	3	Y	61 kDa
			2GJX		Asn157	41.28					
			2GJX		Asn295	120.2					
P07686	Beta-hexosaminidase subunit beta	HEXB_HUMAN	1NOW		Asn84	122.62	556	Y	Y	5	Y	63 kDa
			1NOW		Asn142	48.29					
			1NOW		Asn190	25.5					
			1NOW		Asn327	130.22					
			1NOW		Asn497	37.09					
Q9Y4L1	Hypoxia up-regulated protein 1	HYOU1_HUMAN	N/A				999	Y	N	9	Y	111 kDa
P17936	Insulin-like growth factor-binding protein 3	IBP3_HUMAN	Model on 1h59b	66	Asn116	102.3	291	Y	N	3	Y	32 kDa
Q969P0	Immunoglobulin superfamily member 8	IGSF8_HUMAN	N/A				613	Y	Y	3	Y	65 kDa
Q9Y337	Kallikrein-5	KLK5_HUMAN	2PSX		Asn69	77.48	293	Y	N	4	Y	32 kDa
			2PSX		Asn173	115.47					
			2PSX		Asn208	77.12					
			2PSX		Asn252	133.98					
Q16363	Laminin subunit alpha-4	LAMA4_HUMAN	N/A				1823	Y	N	20	Y	203 kDa
O15230	Laminin subunit alpha-5	LAMA5_HUMAN	N/A				3695	Y	N	25	Y	400 kDa
P07942	Laminin subunit beta-1	LAMB1_HUMAN	Model on 4aqs	93	Asn120	114.53	1786	Y	N	12	Y	198 kDa
			Model on 1kloA	44	Asn356	89.99					
P55268	Laminin subunit beta-2	LAMB2_HUMAN	Model on 4aqsA	68	Asn248	23.47	1798	Y	N	7	Y	196 kDa
			Model on 4aqsA		Asn368	86.45					
P11047	Laminin subunit gamma-1	LAMC1_HUMAN	4AQT		Asn60	122.81	1609	Y	N	14	Y	178 kDa
			4AQT		Asn134	29.31					
Q13753	Laminin subunit gamma-2	LAMC2_HUMAN	N/A				1193	Y	N	6	Y	131 kDa
P01130	Low-density lipoprotein receptor	LDLR_HUMAN	1IJQ		Asn515	136.09	860	Y	Y	5	Y	95 kDa
			1IJQ		Asn657	97.5					
Q99538	Legumain	LGMN_HUMAN	N/A				433	Y	N	5	Y	49 kDa
Q9Y4K0	Lysyl oxidase homolog 2	LOXL2_HUMAN	N/A				774	Y	N	3	Y	87 kDa
Q8NI32	Ly6/PLAUR domain-containing protein 6B	LPD6B_HUMAN	N/A				183	Y	N	1	Y	21 kDa
Q9NS15	Cluster of Latent-transforming growth factor beta-binding protein 3	LTBP3_HUMAN	N/A				1303	Y	N	5	Y	139 kDa
P10253	Lysosomal alpha-glucosidase	LYAG_HUMAN	Model on 3l4ya	44	Asn140	125.92	952	N	Y	7	Y	105 kDa
			Model on 3l4ya		Asn233	17.49					
			Model on 3l4ya		Asn390	24.16					
			Model on 3l4ya		Asn470	34.89					
			Model on 3l4ya		Asn652	69.47					
			Model on 3l4ya		Asn882	75.95					
			Model on 3l4ya		Asn925	91.02					
Q16706	Alpha-mannosidase 2	MA2A1_HUMAN	Model on 3bvxA	41	Asn1125	105.28	1144	N	Y	5	Y	131 kDa
			Model on 3bvxA		Asn1130	27.58					
P08581	Hepatocyte growth factor receptor	MET_HUMAN	3ZCL		Asn1171	59.14	1390	Y	Y	17	Y	156 kDa
			3ZCL		Asn1239	29.81					
P11717	Cation-independent mannose-6-phosphate receptor	MPRI_HUMAN	2V5O		Asn1656	66.28	2491	Y	Y	21	Y	274 kDa
			2V5O		Asn1757	100.52					
			2V5O		Asn1816	86.36					
			2V5O		Asn2085	156.36					
Q8WXI7	Mucin-16	MUC16_HUMAN	N/A				22152	N	Y	46	Y	2353 kDa
P80188	Neutrophil gelatinase-associated lipocalin	NGAL_HUMAN	1X71		Asn85	45.97	198	Y	N	1	Y	23 kDa
Q6UX06	Olfactomedin-4	OLFM4_HUMAN	N/A				510	Y	N	6	Y	57 kDa
P98160	Basement membrane-specific heparan sulfate proteoglycan core protein	PGBM_HUMAN	N/A				4391	Y	N	10	Y	469 kDa
Q02809	Procollagen-lysine,2-oxoglutarate 5-dioxygenase 1	PLOD1_HUMAN	N/A				727	Y	Y	4	Y	84 kDa
Q96NY8	Poliovirus receptor-related protein 4	PVRL4_HUMAN	N/A				510	Y	Y	3	Y	55 kDa
Q92626	Peroxidasin homolog	PXDN_HUMAN	N/A				1479	Y	N	11	Y	165 kDa
O00391	Sulfhydryl oxidase 1	QSOX1_HUMAN	3Q6O		Asn130	149.4	747	Y	Y	4	Y	83 kDa
			3Q6O		Asn243	125.74					
Q13275	Semaphorin-3F	SEM3F_HUMAN	Model on 1q47	54	Asn53	85.05	785	Y	N	7	Y	88 kDa
			Model on 1q47		Asn126	28.49					
Q92673	Sortilin-related receptor	SORL_HUMAN	Model on 1j8e	60	Asn1164	81.91	2214	Y	Y	27	Y	248 kDa
			Model on 1j8e		Asn1191	60.75					
			Model on 2dm4a	92	Asn1706	46.31					
Q99523	Sortilin	SORT_HUMAN	3F6K		Asn98	134.06	831	Y	Y	7	Y	92 kDa
			3F6K		Asn162	99.51					
			3F6K		Asn274	139.63					
			3F6K		Asn406	68.9					
			3F6K		Asn684	127.41					
O43278	Kunitz-type protease inhibitor 1	SPIT1_HUMAN	N/A				529	Y	Y	3	Y	58 kDa
Q9Y5Y6	Suppressor of tumorigenicity 14 protein	ST14_HUMAN	N/A				855	N	Y	4	Y	95 kDa
P52823	Stanniocalcin-1	STC1_HUMAN	N/A				247	Y	N	2	Y	28 kDa
O76061	Stanniocalcin-2	STC2_HUMAN	N/A				302	Y	N	1	Y	33 kDa
Q24JP5	Transmembrane protein 132A	T132A_HUMAN	N/A				1023	Y	Y	3	Y	110 kDa
P24821	Tenascin	TENA_HUMAN	Model on 3tes	58	Asn1079	113.33	2201	Y	N	21	Y	241 kDa
			Model on 3tes		Asn1093	47.51					
			Model on 3tes		Asn1119	98.76					
			Model on 3tes	50	Asn1184	60.08					
			Model on 3tes		Asn1210	109.67					
P61812	Transforming growth factor beta-2	TGFB2_HUMAN	N/A				414	Y	N	3	Y	48 kDa
P49788	Retinoic acid receptor responder protein 1	TIG1_HUMAN	N/A				294	Y	N	1	P	33 kDa
Q9GZM7	Tubulointerstitial nephritis antigen-like	TINAL_HUMAN	N/A				467	Y	N	2	Y	52 kDa
P02787	Serotransferrin	TRFE_HUMAN	4H0W		Asn432	107.27	698	Y	N	2	Y	77 kDa
			4H0W		Asn630	130.6					
Q6EMK4	Vasorin	VASN_HUMAN	N/A				673	Y	Y	5	Y	72 kDa
P25311	Zinc-alpha-2-glycoprotein	ZA2G_HUMAN	1T7V		Asn109	182.94	298	Y	N	4	Y	34 kDa
			1T7V		Asn112	123.92					
			1T7V		Asn128	74.27					
			1T7V		Asn259	88.91					
					**Mean**	**85.44**					
					**SD**	**36.85**					

MCF10A Microsome (Unqiue)

**Acc No**	**Identified Proteins**	**Uniprot ID**	**PDB or Model**	**%matches**	**Asn Site**	**Accessibility**	**Protein Length**	**Signal Peptide**	**Trans-membrane**	**ASN SITE (NO)**	**Glycoprotein**	**Mol Weight**	
P28288	ATP-binding cassette sub-family D member 3	ABCD3_HUMAN	N/A				659	N	Y	1	Y	75 kDa	N = No
O00767	Acyl-CoA desaturase	ACOD_HUMAN	N/A				359	N	Y	3	P	42 kDa	Y = Yes
Q9HD20	Probable cation-transporting ATPase 13A1	AT131_HUMAN	N/A				1204	N	Y	6	Y	133 kDa	P = Potential
Q8WXF7	Atlastin-1	ATLA1_HUMAN	4IDO		Asn177	1.53	558	N	Y	3	P	64 kDa	N/A = Not available
			4IDO		Asn263	74.38							
			4IDO		Asn436	0.8							
Q96G23	Ceramide synthase 2	CERS2_HUMAN	Model on 1x2mA	45	Asn81	76.1	380	N	Y	2	Y	45 kDa	
P52569	Low affinity cationic amino acid transporter 2	CTR2_HUMAN	N/A				658	N	Y	4	Y	72 kDa	
Q53GQ0	Estradiol 17-beta-dehydrogenase 12	DHB12_HUMAN	N/A				312	N	Y	2	P	34 kDa	
P37059	Estradiol 17-beta-dehydrogenase 2	DHB2_HUMAN	N/A				387	Y	Y	1	P	43 kDa	
Q9BW60	Elongation of very long chain fatty acids protein 1	ELOV1_HUMAN	N/A				279	N	Y	2	P	33 kDa	
Q8N766	ER membrane protein complex subunit 1	EMC1_HUMAN	N/A				993	Y	Y	3	Y	112 kDa	
P33947	Cluster of ER lumen protein retaining receptor 2	ERD22_HUMAN	N/A				212	N	Y	1	P	24 kDa	
O75477	Erlin-1	ERLN1_HUMAN	N/A				346	Y	N	1	Y	39 kDa	
O94905	Erlin-2	ERLN2_HUMAN	N/A				339	Y	Y	1	Y	38 kDa	
Q7Z2K6	Endoplasmic reticulum metallopeptidase 1	ERMP1_HUMAN	Model on 3PB6X	40	Asn182	52.21	904	N	Y	4	Y	100 kDa	
A0FGR8-2	Cluster of Isoform 2 of Extended synaptotagmin-2	ESYT2_HUMAN	4NPK		Asn433	46.4	921	N	Y	7	P	99 kDa	
			4NPK		Asn511	78.6							
			4NPK		Asn587	52.63							
			4NPK		Asn643	98.77							
Q53EP0	Fibronectin type III domain-containing protein 3B	FND3B_HUMAN	Model on 1x3dA	62	Asn405	62.52	1204	N	Y	5	P	133 kDa	
P11169	Solute carrier family 2, facilitated glucose transporter member 3	GTR3_HUMAN	N/A				496	N	Y	2	Y	54 kDa	
Q9P035	Very-long-chain (3R)-3-hydroxyacyl-[acyl-carrier protein] dehydratase 3	HACD3_HUMAN	N/A				362	N	Y	3	P	43 kDa	
Q14571	Cluster of Inositol 1,4,5-trisphosphate receptor type 2	ITPR2_HUMAN	Model on 3jrrA	82	Asn82	154.25	2701	N	Y	18	P	308 kDa	
			Model on 3jrrA		Asn103	10.53							
			Model on 3jrrA		Asn215	133.79							
P13473	Lysosome-associated membrane glycoprotein 2	LAMP2_HUMAN	N/A				410	Y	Y	16	Y	45 kDa	
Q14728	Major facilitator superfamily domain-containing protein 10	MFS10_HUMAN	N/A				455	N	Y	1	P	48 kDa	
O14880	Microsomal glutathione S-transferase 3	MGST3_HUMAN	N/A				152	N	Y	1	P	17 kDa	
Q13724	Mannosyl-oligosaccharide glucosidase	MOGS_HUMAN	N/A				837	Y	Y	2	Y	92 kDa	
Q969V3	Nicalin	NCLN_HUMAN	N/A				563	Y	Y	2	Y	63 kDa	
P69849	Nodal modulator 3	NOMO3_HUMAN	N/A				1222	Y	Y	7	Y	134 kDa	
Q9Y639	Neuroplastin	NPTN_HUMAN	Model on 2wv3A	92	Asn171	46.49	398	Y	Y	7	Y	44 kDa	
			Model on 2wv3A		Asn197	89.36							

			Model on 2wv3A		Asn229	72.58					
			Model on 2wv3A		Asn284	77.93					
			Model on 2wv3A		Asn296	71.46					
			Model on 2wv3A		Asn317	32.14					
Q9H0X9	Oxysterol-binding protein-related protein 5	OSBL5_HUMAN	N/A				879	N	Y	3	P	99 kDa
Q92508	Piezo-type mechanosensitive ion channel component 1	PIEZ1_HUMAN	N/A				2521	Y	Y	8	Y	287 kDa
Q8IY17	Neuropathy target esterase	PLPL6_HUMAN	N/A				1366	Y	Y	4	Y	150 kDa
Q96AA3	Protein RFT1 homolog	RFT1_HUMAN	N/A				541	N	Y	1	P	60 kDa
Q8N357	Solute carrier family 35 member F6	S35F6_HUMAN	N/A				371	Y	Y	3	Y	40 kDa
Q92504	Zinc transporter SLC39A7	S39A7_HUMAN	N/A				469	N	Y	1	P	50 kDa
Q9H2J7	Sodium-dependent neutral amino acid transporter B(0)AT2	S6A15_HUMAN	N/A				730	N	Y	5	Y	82 kDa
O15126	Secretory carrier-associated membrane protein 1	SCAM1_HUMAN	N/A				338	N	Y	2	P	38 kDa
Q9UGP8	Translocation protein SEC63 homolog	SEC63_HUMAN	N/A				760	N	Y	2	P	88 kDa
P35610	Sterol O-acyltransferase 1	SOAT1_HUMAN	N/A				550	N	Y	3	P	65 kDa
O15269	Serine palmitoyltransferase 1	SPTC1_HUMAN	N/A				473	N	Y	1	P	53 kDa
O43291	Kunitz-type protease inhibitor 2	SPIT2_HUMAN	Model on 4isoB	59	Asn57	31.32	252	Y	Y	2	Y	28 kDa
P43307	Translocon-associated protein subunit alpha	SSRA_HUMAN	N/A				286	Y	Y	2	Y	32 kDa
Q8TCJ2	Dolichyl-diphosphooligosaccharide--protein glycosyltransferase subunit STT3B	STT3B_HUMAN	N/A				826	N	Y	6	P	94 kDa
Q03518	Antigen peptide transporter 1	TAP1_HUMAN	N/A				808	N	Y	5	P	87 kDa
Q9NZ01	Very-long-chain enoyl-CoA reductase	TECR_HUMAN	N/A				308	N	Y	3	Y	36 kDa
Q6NUQ4	Transmembrane protein 214	TM214_HUMAN	N/A				689	N	Y	4	Y	77 kDa
Q9HD45	Transmembrane 9 superfamily member 3	TM9S3_HUMAN	N/A				589	Y	Y	2	Y	68 kDa
Q92544	Transmembrane 9 superfamily member 4	TM9S4_HUMAN	N/A				642	Y	Y	1	P	75 kDa
Q9Y320	Thioredoxin-related transmembrane protein 2	TMX2_HUMAN	Model on 2dj0A	99	Asn165	132.71	296	Y	Y	2	P	34 kDa
			Model on 2dj0A		Asn185	29.89					
Q93050	V-type proton ATPase 116 kDa subunit a isoform 1	VPP1_HUMAN	N/A				837	N	Y	4	P	96 kDa
					**Mean**	**64.84**					
					**SD**	**40.97**					

MCF10A Cell surface (Unique)

Acc No	Identified Proteins	Uniprot ID	PDB or Model	%matches	Asn Site	Accessibility	Protein Length	Signal Peptide	Transmembrane	ASN SITE (NO)	Glycoprotein	Mol Weight	
Q96S66	Chloride channel CLIC-like protein 1	CLCC1_HUMAN	N/A				551	Y	Y	1	Y	62 kDa	N = No
Q9UBS4	DnaJ homolog subfamily B member 11	DJB11_HUMAN	N/A				358	Y	N	2	Y	41 kDa	Y = Yes
Q14534	Squalene monooxygenase	ERG1_HUMAN	N/A				574	N	Y	1	P	64 kDa	P = Potential
O95302	Peptidyl-prolyl cis-trans isomerase FKBP9	FKBP9_HUMAN	Model on 2pbcA	53	Asn286	38.5	570	Y	N	4	Y	63 kDa	N/A = Not available
			Model on 2pbcA		Asn302	103.61							
Q8NBJ5	Procollagen galactosyltransferase 1	GT251_HUMAN	N/A				622	Y	N	3	Y	72 kDa	
P07099	Epoxide hydrolase 1	HYEP_HUMAN	N/A				455	Y	Y	2	P	53 kDa	
Q9NPH3	Interleukin-1 receptor accessory protein	IL1AP_HUMAN	4DEP		Asn57	73.52	570	Y	Y	7	Y	65 kDa	
			4DEP		Asn107	116.34							
			4DEP		Asn111	61.15							
			4DEP		Asn118	55.44							
			4DEP		Asn196	53.88							
			4DEP		Asn209	70.53							
			4DEP		Asn299	102.99							
P56199	Integrin alpha-1	ITA1_HUMAN	1PT6		Asn217	111.34	1179	Y	Y	26	Y	131 kDa	
			1PT6		Asn317	139.22							
			1PT6		Asn341	82.09							
Q9UIQ6	Leucyl-cystinyl aminopeptidase	LCAP_HUMAN	N/A				1025	Y	Y	18	Y	117 kDa	
P15529	Cluster of Membrane cofactor protein	MCP_HUMAN	3O8E		Asn83	130.71	392	Y	Y	3	Y	44 kDa	
			3O8E		Asn114	53.41							
			3O8E		Asn273	110.84							
Q9H8H3	Methyltransferase-like protein 7A	MET7A_HUMAN	N/A				244	Y	N	1	P	28 kDa	
P08581	Hepatocyte growth factor receptor	MET_HUMAN	3ZCL		Asn1171	59.14	1390	Y	Y	17	Y	156 kDa	
			3ZCL		Asn1239	29.81							
Q9UNW1	Multiple inositol polyphosphate phosphatase 1	MINP1_HUMAN	N/A				487	Y	Y	2	Y	55 kDa	
O95297	Myelin protein zero-like protein 1	MPZL1_HUMAN	Model on 1neuA	43	Asn50	66.93	269	Y	Y	3	Y	29 kDa	
			Model on 1neuA		Asn131	15.78							
Q6PIU2	Neutral cholesterol ester hydrolase 1	NCEH1_HUMAN	Model on 3ainA	40	Asn270	8.01	24.005	72.015	408	Y	Y	3	
			Model on 3ainA		Asn287	42.73							
O15118	Niemann-Pick C1 protein	NPC1_HUMAN	3GKH		Asn70	132.02	1278	Y	Y	19	Y	142 kDa	
			3GKH		Asn121	81.95							
			3GKH		Asn135	103.42							
			3GKH		Asn185	66.36							
			3GKH		Asn222	66.36							
Q92823	Neuronal cell adhesion molecule	NRCAM_HUMAN	Model on 3p3yA	56	Asn83	78.39	1304	Y	Y	20	Y	144 kDa	
			Model on 3p3yA		Asn223	51.84							
			Model on 3p3yA		Asn245	62.91							
			Model on 3p3yA		Asn251	54.56							
			Model on 3p3yA		Asn276	112.44							
			Model on 3p3yA		Asn314	145.96							
			Model on 3p3yA		Asn433	48.17							
			Model on 1ueY	98	Asn673	40.25							
			Model on 1ueY		Asn716	5.13							
Q32P28	Prolyl 3-hydroxylase 1	P3H1_HUMAN	N/A				736	Y	N	3	Y	83 kDa	
P13674	Prolyl 4-hydroxylase subunit alpha-1	P4HA1_HUMAN	4BTB		Asn113	42.35	534	Y	N	2	Y	61 kDa	
O15460	Prolyl 4-hydroxylase subunit alpha-2	P4HA2_HUMAN	Model on 4bt9	63	Asn115	45.81	535	Y	N	2	Y	61 kDa	
Q9H7Z7	Prostaglandin E synthase 2	PGES2_HUMAN	Model on 1z9hA	99	Asn163	34.64	377	N	Y	1	P	42 kDa	
O00469	Procollagen-lysine,2-oxoglutarate 5-dioxygenase 2	PLOD2_HUMAN	N/A				737	Y	Y	7	Y	85 kDa	
Q9UIW2	Plexin-A1	PLXA1_HUMAN	Model on 3ig3A	49	Asn1341	0.14	1896	Y	Y	16	Y	211 kDa	
			Model on 3ig3A		Asn1559	48.75							
Q15293	Reticulocalbin-1	RCN1_HUMAN	N/A				331	Y	N	1	Y	39 kDa	
Q8TC12	Retinol dehydrogenase 11	RDH11_HUMAN	Model on 3rd5A	47	Asn174	1.62	318	Y	Y	2	P	35 kDa	
			Model on 3rd5A		Asn298	52.4							
Q00765	Receptor expression-enhancing protein 5	REEP5_HUMAN	N/A				189	Y	Y	1	Y	21 kDa	
Q9NTJ5	Phosphatidylinositide phosphatase SAC1	SAC1_HUMAN	N/A				587	N	Y	5	P	67 kDa	
Q8WTV0-2	Isoform 1 of Scavenger receptor class B member 1	SCRB1_HUMAN	N/A				552	Y	Y	9	Y	57 kDa	
P50454	Serpin H1	SERPH_HUMAN	Model on 3zhaA	99	Asn120	113.05	418	Y	N	2	Y	46 kDa	
			Model on 3zhaA		Asn125	64.4							
Q9H173	Nucleotide exchange factor SIL1	SIL1_HUMAN	N/A				461	Y	N	2	Y	52 kDa	
O43278	Kunitz-type protease inhibitor 1	SPIT1_HUMAN	N/A				529	Y	Y	3	Y	58 kDa	
Q8NFQ8	Torsin-1A-interacting protein 2	TOIP2_HUMAN	N/A				470	N	Y	1	Y	51 kDa	
O14763	Tumor necrosis factor receptor superfamily member 10B	TR10B_HUMAN	N/A				440	Y	Y	1	P	48 kDa	
Q9Y6I9	Testis-expressed sequence 264 protein	TX264_HUMAN	N/A				313	Y	N	1	Y	34 kDa	
Q9NYU2	UDP-glucose:glycoprotein glucosyltransferase 1	UGGG1_HUMAN	N/A				1555	Y	Y	3	Y	177 kDa	
Q9Y6M5	Zinc transporter 1	ZNT1_HUMAN	N/A				507	N	Y	2	Y	55 kDa	
					**Mean**	**67.70**							
					**SD**	**37.66**							

MCF10A secreted (Unique)

Acc No	Identified Proteins	Uniprot ID	PDB or Model	%matches	Asn Site	Accessibility	Protein Length	Signal Peptide	Transmembrane	ASN SITE (NO)	Glycoprotein	Mol Weight	
P01009	Alpha-1-antitrypsin	A1AT_HUMAN	2QUG		Asn70	114.59	418	Y	N	3	Y	47 kDa	N = No
			2QUG		Asn107	70.94							Y = Yes
			2QUG		Asn271	20.88							P = Potential
P01023	Cluster of Alpha-2-macroglobulin	A2MG_HUMAN	4ACQ		Asn55	146.06	1474	Y	N	8	Y	163 kDa	N/A = Not available
			4ACQ		Asn70	95.14							
			4ACQ		Asn247	96.85							
			4ACQ		Asn396	108.31							
			4ACQ		Asn410	91.42							
			4ACQ		Asn869	101.89							
			4ACQ		Asn991	72.29							
			4ACQ		Asn1424	114.32							
P01011	Alpha-1-antichymotrypsin	AACT_HUMAN	1AS4		Asn93	112.72	423	Y	N	6	Y	48 kDa	
			1AS4		Asn106	88.7							
			1AS4		Asn127	76.52							
			1AS4		Asn186	123.36							
			1AS4		Asn271	86.61							
O14672	Disintegrin and metalloproteinase domain-containing protein 10	ADA10_HUMAN	Model on 2ao7A	92	Asn551	71.72	748	Y	Y	4	Y	84 kDa	
O00468-6	Isoform 6 of Agrin	AGRIN_HUMAN	Model on 1jb3	90	Asn135	54.9	2067	Y	Y	4	Y	215 kDa	
O75882	Attractin	ATRN_HUMAN	Model on 1kloA	40	Asn1073	162.04	1429	N	Y	26	Y	159 kDa	
			Model on 1kloA		Asn1082	127.44							
P13497	Bone morphogenetic protein 1	BMP1_HUMAN	3EDH		Asn91	109.12	986	Y	N	5	Y	111 kDa	
P00736	Complement C1r subcomponent	C1R_HUMAN	1GPZ		Asn514	128.19	705	Y	N	4	Y	80 kDa	
			1GPZ		Asn581	139.62							
P09871	Complement C1s subcomponent	C1S_HUMAN	1ELV		Asn406	124.37	688	Y	N	3	Y	77 kDa	
			1ELV		Asn682	51.82							
Q9BRK5	45 kDa calcium-binding protein	CAB45_HUMAN	N/A				362	Y	Y	1	Y	42 kDa	
P19022	Cadherin-2	CADH2_HUMAN	Model on 3q2w	98	Asn190	103.74	906	Y	Y	9	Y	100 kDa	
			Model on 3q2w		Asn273	125.53							
			Model on 3q2w		Asn325	77.63							
			Model on 3q2w		Asn402	82.43							

			Model on 3q2w		Asn572	73.26						
			Model on 3q2w		Asn622	71.7						
			Model on 3q2w		Asn651	46.13						
			Model on 3q2w		Asn692	52.53						
P53634	Dipeptidyl peptidase 1	CATC_HUMAN	3PDF		Asn29	101.28	463	Y	N	4	P	52 kDa
			3PDF		Asn53	87.11						
			3PDF		Asn119	85.83						
			3PDF		Asn276	112.51						
P07711	Cathepsin L1	CATL1_HUMAN	1CS8		Asn221	69.15	333	Y	N	1	Y	38 kDa
P08603	Complement factor H	CFAH_HUMAN	2WII		Asn217	56.56	1231	Y	N	9	Y	139 kDa
P02462	Collagen alpha-1(IV) chain	CO4A1_HUMAN	N/A				1669	Y	N	4	Y	161 kDa
Q02388	Collagen alpha-1(VII) chain	CO7A1_HUMAN	N/A				2944	Y	N	4	Y	295 kDa
Q99715	Collagen alpha-1(XII) chain	COCA1_HUMAN	N/A				3063	Y	N	9	Y	333 kDa
Q9H3G5	Probable serine carboxypeptidase CPVL	CPVL_HUMAN	N/A				476	Y	N	4	Y	54 kDa
P09603	Macrophage colony-stimulating factor 1	CSF1_HUMAN	1HMC		Asn154	56.76	554	Y	Y	4	Y	60 kDa
			1HMC		Asn172	57.61						
P13611	Versican core protein	CSPG2_HUMAN	N/A				3396	Y	N	24	Y	373 kDa
O94985-2	Isoform 2 of Calsyntenin-1	CSTN1_HUMAN	N/A				981	Y	Y	4	Y	109 kDa
O94907	Dickkopf-related protein 1	DKK1_HUMAN	N/A				266	Y	N	1	Y	29 kDa
Q14517	Protocadherin Fat 1	FAT1_HUMAN	N/A				4588	Y	Y	29	Y	506 kDa
Q9NYQ8	Protocadherin Fat 2	FAT2_HUMAN	N/A				4349	Y	Y	41	Y	479 kDa
P23142	Cluster of Fibulin-1	FBLN1_HUMAN	N/A				703	Y	N	3	Y	77 kDa
Q12805	EGF-containing fibulin-like extracellular matrix protein 1	FBLN3_HUMAN	N/A				493	Y	N	1	Y	55 kDa
P35556	Fibrillin-2	FBN2_HUMAN	N/A				2912	Y	N	12	Y	315 kDa
Q12841	Follistatin-related protein 1	FSTL1_HUMAN	N/A				308	Y	N	3	Y	35 kDa
Q99988	Growth/differentiation factor 15	GDF15_HUMAN	N/A				308	Y	N	2	Y	34 kDa
P07093	Glia-derived nexin	GDN_HUMAN	4DY0		Asn118	54.02	398	Y	Y	2	Y	44 kDa
			4DY0		Asn159	115.07						
Q92820	Gamma-glutamyl hydrolase	GGH_HUMAN	1L9X		Asn116	70.56	318	Y	N	4	Y	36 kDa
			1L9X		Asn163	66.09						
			1L9X		Asn203	43.44						
			1L9X		Asn307	93.36						
P35052	Glypican-1	GPC1_HUMAN	4ACR		Asn79	103.6	558	Y	Y	2	Y	62 kDa
			4ACR		Asn116	73.03						
P24592	Insulin-like growth factor-binding protein 6	IBP6_HUMAN	Model on 3fcs	53	Asn48	95.63	240	Y	N	1	Y	25 kDa
			Model on 3fcs		Asn97	2.72						
			Model on 3fcs		Asn260	135.49						
			Model on 3fcs		Asn387	30.46						
			Model on 3fcs		Asn396	142.45						
			Model on 3fcs		Asn463	99.28						
			Model on 3fcs		Asn471	31.78						
			Model on 3fcs		Asn541	61.38						
			Model on 3fcs		Asn575	59.2						
			Model on 2kv9	66	Asn771	87.37						
Q13007	Interleukin-24	IL24_HUMAN	N/A				206	Y	N	3	Y	24 kDa
P08476	Inhibin beta A chain	INHBA_HUMAN	N/A				426	Y	N	1	Y	47 kDa
P32004	Neural cell adhesion molecule L1	L1CAM_HUMAN	N/A				1257	Y	Y	22	Y	140 kDa
P50851	Lipopolysaccharide-responsive and beige-like anchor protein	LRBA_HUMAN	1t77		Asn2210	53.27	2863	N	Y	15	P	319 kDa
Q86X29	Lipolysis-stimulated lipoprotein receptor	LSR_HUMAN	N/A				649	N	Y	2	P	71 kDa
Q14766	Latent-transforming growth factor beta-binding protein 1	LTBP1_HUMAN	Model on 1lmjA	50	Asn1197	120.57	1721	Y	N	8	Y	187 kDa
			Model on 1ksqA	99	Asn1366	87.73						
P14780	Matrix metalloproteinase-9	MMP9_HUMAN	1L6J		Asn38	128.01	707	Y	N	3	Y	78 kDa
			1L6J		Asn120	68.13						
			1L6J		Asn127	50.26						
Q13421-2	Isoform 3 of Mesothelin	MSLN_HUMAN	N/A				630	Y	N	4	Y	71 kDa
Q04721	Neurogenic locus notch homolog protein 2	NOTC2_HUMAN	2F8Y		Asn1922	89.22	2471	Y	Y	9	Y	265 kDa
O14786	Neuropilin-1	NRP1_HUMAN	2QQM		Asn150	83.36	923	Y	Y	6	Y	103 kDa
			2QQM		Asn261	60.27						
			2QQM		Asn300	84.55						
			2QQM		Asn522	95.01						
P05121	Plasminogen activator inhibitor 1	PAI1_HUMAN	1LJ5		Asn232	26.69	402	Y	N	3	Y	45 kDa
			1LJ5		Asn288	95.3						
			1LJ5		Asn352	60.77						
P07585	Decorin	PGS2_HUMAN	Model on 1xku	92	Asn211	48.49	359	Y	N	3	Y	40 kDa
			Model on 1xku		Asn262	131.33						
			Model on 1xku		Asn303	80.32						
Q92626	Peroxidasin homolog	PXDN_HUMAN	N/A				1479	Y	N	11	Y	165 kDa
O00391	Sulfhydryl oxidase 1	QSOX1_HUMAN	3Q6O		Asn130	149.4	747	Y	Y	4	Y	83 kDa
			3Q6O		Asn243	125.74						
P07602	Proactivator polypeptide	SAP_HUMAN	2DOB		Asn80	134.59	524	Y	N	5	Y	58 kDa
			2DOB		Asn101	107.63						
			1N69		Asn215	68.44						
			2GTG		Asn332	135.24						
			3BQP		Asn425	94.32						
O95486	Protein transport protein Sec24A	SC24A_HUMAN	2NUT		Asn485	46.04	1093	N	Y	8	P	120 kDa
			2NUT		Asn585	84.17						
			2NUT		Asn951	96.79						
			2NUT		Asn959	120.28						
			2NUT		Asn999	100.14						
Q9H4F8	SPARC-related modular calcium-binding protein 1	SMOC1_HUMAN	N/A				434	Y	N	3	Y	48 kDa
P36952	Serpin B5	SPB5_HUMAN	1WZ9		Asn99	118.16	375	Y	N	4	Y	42 kDa
			1WZ9		Asn133	18.77						
			1WZ9		Asn188	6.71						
			1WZ9		Asn361	48.95						
Q24JP5	Transmembrane protein 132A	T132A_HUMAN	N/A				1023	Y	Y	3	Y	110 kDa
P01033	Metalloproteinase inhibitor 1	TIMP1_HUMAN	3V96		Asn101	83.1	207	Y	N	2	Y	23 kDa
O95407	Tumor necrosis factor receptor superfamily member 6B	TNF6B_HUMAN	3K51		Asn173	96.69	300	Y	N	1	Y	33 kDa
P00749	Urokinase-type plasminogen activator	UROK_HUMAN	2O8T		Asn322	110.37	431	Y	N	1	Y	49 kDa
					**Mean**	**86.56**						
					**SD**	**33.54**						

Supplementary Table 3A

Subcellular fraction	Proportion of putative *N*-glycoproteins in subcellular proteomes (Number of putative *N*-glycoproteins / Number of total proteins)		
	MCF7 Total proteins = 2297	MDA468 Total proteins = 2636	MCF10A Total proteins = 2042
Microsome	22.7% (346/1525)	15.7% (313/1988)	24.1% (337/1399)
Cell surface	15.7% (94/599)	31.0% (45/145)	25.7% (105/490)
Secreted	24.7% (302/1222)	22.4% (302/1348)	19.6% (187/952)

Supplementary Table 3B

Subcellular fraction	Sequon-weighted normalized spectral count of top-100 most abundant putative *N*-glycoproteins / Total putative *N*-glycoproteins		
	MCF7	MDA468	MCF10A
Microsome	26.0/36.9 (70.5%)	17.0/23.6 (72.0%)	20.8/29.4 (70.7%)
Cell surface	7.2/7.2 (100%)	8.9/8.9 (100%)	6.7/6.8 (99.0%)
Secreted	41.3/51.1 (77.5%)	21.8/28.6 (76.2%)	22.1/24.8 (89.1%)

Supplementary Table 4

Cell line	Subcellular fraction	Degree of terminal *N*-glycan determinants / total complex/hybrid type *N*-glycans (%)		
		β-galactosylation	α1,6-fucosylation	α-sialylation
MCF7	Microsome	66.30 ± 2.06	86.47 ± 2.10	38.14 ± 1.41
	Cell surface	91.23 ± 1.66	85.02 ± 4.91	38.15 ± 5.01
	Secreted	68.43 ± 0.25	78.24 ± 1.15	57.55 ± 2.37
	R^2	0.05	(-) 0.61	0.64
MDA468	Microsome	48.36 ± 0.70	60.50 ± 7.59	67.28 ± 3.54
	Cell surface	78.01 ± 0.32	68.62 ± 0.92	69.85 ± 1.92
	Secreted	26.89 ± 2.33	58.59 ± 1.37	94.99 ± 0.30
	R^2	0.00	0.04	0.41
MCF10A	Microsome	46.49 ± 0.61	68.98 ± 1.68	83.77 ± 0.78
	Cell surface	49.12 ± 1.68	75.81 ± 1.07	85.02 ± 1.91
	Secreted	55.98 ± 5.54	82.23 ± 1.89	77.98 ± 3.78
	R^2	0.64	0.81	(-) 0.65
